# Supplementary material for: Small-angle x-ray and neutron scattering of MexR and its complex with DNA supports a conformational selection binding model
Source: Biophys J. 2022 Dec 5;122(2):408–18. doi: 10.1016/j.bpj.2022.11.2949 (PMC9892617; doi:10.1016/j.bpj.2022.11.2949)
Supplement: Document S1. Figures S1–S12 and Tables S1 and S2 [file mmc1.pdf]

Biophysical Journal, Volume *122*

Supplemental information

Small-angle x-ray and neutron scattering of MexR and its complex with DNA supports a conformational selection binding model

Francesca Caporaletti, Zuzanna Pietras, Vivian Morad, Lars-Göran Mårtensson, Frank Gabel, Björn Wallner, Anne Martel, and Maria Sunnerhagen

SUPPLEMENTARY MATERIAL OF SMALL-ANGLE X-RAY AND NEUTRON SCATTERING OF MEXR AND ITS COMPLEX WITH DNA SUPPORTS A CONFORMATIONAL SELECTION BINDING MODEL

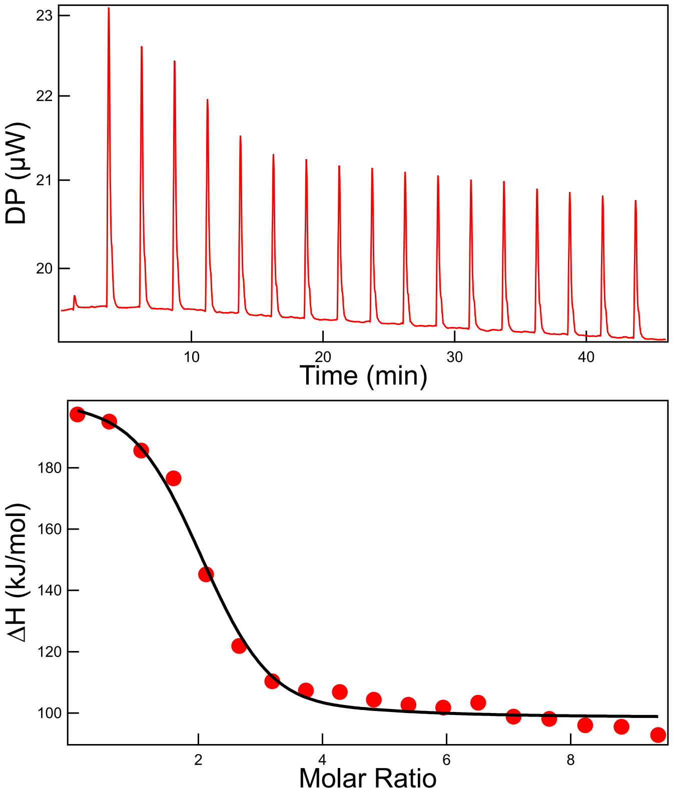

K_D $(10^{-9} M)$	N_s	ΔH (kJ/mol)	ΔG (kJ/mol)	$-T\Delta S$ (kJ/mol)
240 ±60	1.91 ±0.06	105 ±5	-37.2	-142

Figure S1: **ITC profiles for the binding of MexR to PII DNA.** The upper panel presents the thermogram for MexR titration into double stranded PII DNA solution. The bottom panel shows the heat evolution of the added MexR to PII DNA. Data (red circles) were fitted to the "one set of sites" model, and the solid black line represents the best fit. The table reports the thermodynamic parameters for the association of MexR with the PII DNA segment. Each column represents the result of the fit: K_D: dissociation constant, N_s: number of sites, ΔH: enthalpy, ΔG: free enthalpy of Gibbs, -TΔS: entropy

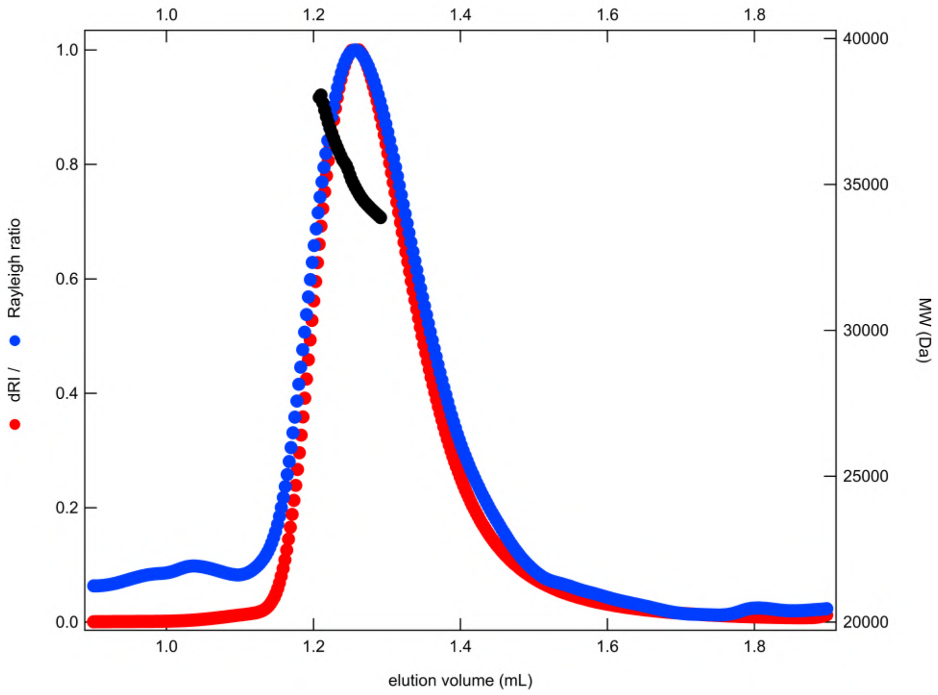

Figure S2: **The MALLS (blue) and dRI (red) traces acquired for the SEC of MexR protein.** The MW distribution through the MexR peak is shown as a black line

Table S1: Resume table of results and conduction for apo-MexR. The tables are inspired by the guidelines described in Trewhella *et at.* (26).

SAMPLE	apo-MexR
**ORGANISM**	*P. aeruginosa*
**SOURCE**	produced at LiU
**UNIPROT ID**	P52003
**AMINO ACID SEQUENCE**	SM VNPDLMPALMAVFQHVRTRIQ SELDCQRLDLTPPDVHVLKLIDEQRGLNLQDLGRQMCRDKAL ITRKIRELEGRNLVRRERNP SDQRSFQLFLTDEGLAIHQHAEAIMSRVHDELFAPLTPVEQATL VHLLDQCL
**EXTINCTION COEFFICIENT AT 280 nm** ($M^{-1}cm^{-1}$)	8.00 10^{-3}
**PARTIAL SPECIFIC VOLUME** (cm^3g^{-1})	0.74
**STOICHIOMETRY**	2
**MONOMER VOLUME FROM CHEMICAL COMPOSITION** (Å^3)	18600
**MONOMER MASS FROM CHEMICAL COMPOSITION (KDa)**	15.3
**SOLVENT COMPOSITION**	20 mM HEPES, pH 7.1, 150 mM NaCl, 10 mM DTT, 1% v/v glycerol
**SLD SAMPLE** (10^{-6}**Å**$^{-2}$)	12.3
**SLD SOLVENT** (10^{-6}**Å**$^{-2}$)	9.4
**CONTRAST** (10^{-6}**Å**$^{-2}$)	2.9

Table S1 continued from previous page

SAMPLE	apo-MexR
**SEC COLUMN**	S75 Increase 5/150
**LOADING CONCENTRATION** ($mg\,ml^{-1}$)	8.5
**INJECTION VOLUME**	35 μl
**FLOW RATE** ($ml\,min^{-1}$)	0.35
**SAXS data collection parameters**	
**SOURCE**	Petra III U29 undulator
**WAVELENGTH**	1.24 Å
**BEAM GEOMETRY AT SAMPLE**	0.99 mm capillary
**BEAM SIZE AT THE DETECTOR**	0.2 × 0.05 mm^2
**DETECTOR**	Pilatus 6M
**SAMPLE TO DETECTOR DISTANCE (m)**	3 (position 2)
**q-RANGE** (Å^{-1})	0.0024–0.73
**EXPOSURE TIME/NUMBER OF FRAMES**	1s/2880
**SAMPLE TEMPERATURE**	20 °C
**MONITORING FOR RADIATION DAMAGE**	SEC-SAXS
**MALLS**	Wyatt Technologies Mini-Dawn TREOS with an in-built quasi elastic light scattering (QELS) module coupled to an OptiLab T-Rex refractometer
**RI**	0.185 $ml\,g^{-1}$
**Software**	
**SAMPLE INJECTION**	ChemStation/BECQUEREL
**SAS DATA REDUCTION**	The SASFLOW pipeline incorporating RADAVER from the ATSAS 3.0 suite and CHROMIXS from the ATSAS 3.0
**CALCULATION OF** ϵ **FROM SEQUENCE**	EXPASY
**CALCULATION OF SLD FROM CHEMICAL COMPOSITION**	MULCh from The University of Sydney (39)
**STRUCTURAL PARAMETER**	$P(r)$ inversion with ATASAS/GNOM<br>Guinier analysis with PRIMUS;<br>Porod volume: GNOM
**BEAD MODELING**	DAMMIF
**ATOMIC STRUCTURE MODELING**	Molecular dynamics with Gromacs 4.5.5
**MODELING OF MISSING SEQUENCE FROM PDB FILE**	Modeller 9.13
**MOLECULAR GRAPHICS**	CHIMERA USFC
**Structural parameter**	
**GUINIER ANALYSIS (RECIPROCAL SPACE)**	
**I(0)** (cm^{-1})	0.0066 ± 0.0002
R_g **(Å)**	23.21 ± 0.12
sR_g **RANGE**	0.25 - 1.30
**FIDELITY**	0.2
**MW FROM ATSAS (Qp) (Da)**	26900
**MW FROM ATSAS (MoW) (Da)**	24700
**MW FROM ATSAS (Vc) (Da)**	32100
**MW FROM ATSAS (Size and Shape) (Da)**	34700
**MW FROM ATSAS (Bayesian Inference) (Da)**	31700
**MW FROM MALLS (kDa)**	34-38
**MW EXPECTED VALUE, DIMER (kDa)**	30.6
***P(r)* ANALYSIS (REAL SPACE)**	
**I(0)** (cm^{-1})	0.0067 ± 0.0002
**APPARENT** R_g **(Å)**	23.4
D_{max} **(Å)**	77 ± 5

Table S1 continued from previous page

SAMPLE	apo-MexR
**q-RANGE (Å^{-1})**	0.010 - 0.34
**GNOM QUALITY**	0.95
**POROD VOLUME (Å)**	55700

Table S2: Resume table of results and conduction for MexR-PII. The tables are inspired by the guidelines described in J. et al. [26].

SAMPLE	MexR-PII					
	dMexR-PII 0%D_2O	dMexR-PII 56%D_2O	dMexR-PII 89%D_2O	hMexR-PII 0%D_2O	hMexR-PII 79%D_2O	MexR-PII
**ORGANISM**	*Pseudomonas aeruginosa*					
**SOURCE**	Protein: produced at LiU: both hydrogenated and 73% deuterated with *E. Coli* DNA:EUROGENETEC: SON: 1000831043					
**UNIPROT ID**	Protein: P52003					
**SEQUENCE NUCLEIC COMPONENT**	FW: 5' - CTT ATT TTA GTT GAC CTT ATC AAC CTT GTT TCA G - 3' RV: 5' - CTG AAA CAA GGT TGA TAA GGT CAA CTA AAA TAA G - 3'					
**AMINO ACID SEQUENCE**	SM VNPDLMPALMAVFQHVRTRIQSELD CQRLDLTPPDVHVLKLIDEQRGLNLQDLGRQMCRDKAL ITRKIRELEGRNLVRRERNP SDQRSFQLFLTDEGLAIHQHAEAIMSRVHDELFAPLTPVEQATL VHLLDQCL					
**EXTINCTION COEFFICIENT** ($mL\,mg^{-1}cm^{-1}$)	Protein: 8.0010^{-3}(280 nm) DNA: 25.4 (260nm)					
**PARTIAL SPECIFIC VOLUME** ($cm^3 g^{-1}$)	0.681					
**STOICHIOMETRY**	2 MexR: 1RV: 1FW					
**COMPLEX VOLUME FROM CHEM. COMPOSITION (Å^3)**	60200					
**COMPLEX MASS FROM CHEM. COMPOSITION (Da)**	52120.84					
**CONCENTRATION (mg ml^{-1})**	6 ± 1					3.1 ± 0.7
**SOLVENT COMPOSITION**	20 mM sodium phosphate buffer pH=7.1; 150 mM NaCl; 10 mM DTT					
**SLD SAMPLE** (10^{-6} **Å**$^{-2}$)	4.50	5.11	5.47	2.29	3.14	13.1
**SLD SOLVENT** (10^{-6} **Å**$^{-2}$)	-0.560	3.33	5.62	-0.560	4.93	9.40
**CONTRAST** (10^{-6}**Å**$^{-2}$)	5.06	1.78	-0.157	2.85	-1.79	3.74
**CONTRAST PROTEIN** (10^{-6} **Å**$^{-2}$)	5.74	2.59	0.74	2.36	-2.08	2.85
**CONTRAST DNA** (10^{-6} **Å**$^{-2}$)	3.78	0.23	-1.89	3.79	-1.86	5.45
**SAS data collection parameters**						
**EXPERIMENTAL SOURCE**	D22 at ILL					SAXess (Anton Paar)
**WAVELENGTH (Å)**	6 ± 0.6					0.15418
**BEAM GEOMETRY AT SAMPLE (mm)**	7 x10					10, slit
**SOURCE GEOMETRY (mm)**	40 x 55					10, slit

Table S2 continued from previous page

SAMPLE	MexR-PII					
	dMexR-PII 0%D_2O	dMexR-PII 56%D_2O	dMexR-PII 89%D_2O	hMexR-PII 0%D_2O	hMexR-PII 79%D_2O	MexR-PII
**COLLIMATOR L (m): SAMPLE DETECTOR D (m)**	1st: 5.6 : 5.6 2nd: 1.6 : 2.8					0.5 : 0.5
**q-RANGE (Å^{-1})**	0.014 - 0.5					0.014 - 0.5
**ABSOLUTE SCALING METHOD**	Direct: normalisation by flux at the sample position					Normalisation over empty beam peak
**BASIS FOR NORMALISATION TO CONSTANT COUNT**	Normalisation to monitor count					
**EXPOSURE TIME (min)**	41<br>15	31<br>15	71<br>30	41<br>30	42<br>15	4 x 20
**SAMPLE CONFIGURATION INCLUDING PATH LENGTH**	in 1 mm thickness Suprasil Quartz rectangular Hellma cuvettes					capillary diameter=1mm
**SAMPLE TEMPERATURE**	10 °C					
**Software**						
**SAS DATA REDUCTION**	GRASP and NCNR Macro for IGOR					SAXS quant 1D
**CALCULATION OF ϵ FROM SEQUENCE**	EXPASY for protein and OLIGOCALC for the DNAs					
**CALCULATION OF ρ FROM CHEMICAL COMPOSITION**	BSLDC from ISIS: http://psldc.isis.rl.ac.uk/Psldc<br>MULCH from the University of Sydney: http://smb-research.smb.usyd.edu.au/NCVWeb/ (39)					
**STRUCTURAL PARAMETERS**	*P(r)* inversion using ATSAS/GNOM;<br>Guinier analysis using ATSAS/Primus and NCNR macro;<br>Porod Volume using ATSAS/GNOM					
**BEAD MODELING**	ATSAS/MONSA					
**ATOMIC STRUCTURE MODELING**	Molecular Dynamics with Gromacs 4.5.5					
**MODELING OF MISSING SEQUENCE FROM PDB FILE**	Modeller 9.13					
**MOLECULAR GRAPHICS**	UCSF Chimera					
**Structural parameters**						
**GUINIER ANALYSIS (RECIPROCAL SPACE)[1]**						
**$I(0)_{theorical\ 1mg/ml}$ (cm^{-1})**	0.098	0.0061	0.0017	0.034	0.013	0.052
**$I(0)_{theorical}$ experimental concentration (cm^{-1})**	0.59	0.037	0.010	0.21	0.078	0.16
**I(0) (cm^{-1})**	0.66±0.01	0.038 ± 0.0008	0.010 ± 0.0005	0.21 ± 0.0009	0.081 ± 0.0007	0.191 ± 0.005
**R_g (Å)**	26.4 ± 0.5	23.7 ± 0.6	22 ± 2	28.9 ± 0.2	19.9 ± 0.4	27.7 ± 0.8
qR_g	0.50 - 1.3	0.44 - 1.3	0.46 - 1.2	0.51 - 1.3	0.45 - 1.3	0.48 - 1.3
χ^2	0.11	0.059	0.099	1.1	0.29	0.11
**MW FROM I(0) (Da)**	55600	28200	20800	54200	53400	52400
**MW EXPECTED FROM THE CHEM. COMPOSITION (Da)**	52200	31700		52200	52200	52200
***P(r)* ANALYSIS (REAL SPACE)**						

[1]This part is done with the NCNR IGOR Macro (34)

Table S2 continued from previous page

SAMPLE	MexR-PII					
	dMexR-PII 0% D_2O	dMexR-PII 56% D_2O	dMexR-PII 89% D_2O	hMexR-PII 0% D_2O	hMexR-PII 79% D_2O	MexR-PII
**I(0)** (cm^{-1})	0.680 ± 0.008	0.0383 ± 0.0006	0.0087 ± 0.0003	0.213 ± 0.001	0.081 ± 0.007	0.191 ± 0.002
**apparent R_g (Å)**	26.9 ± 0.6	24.1 ± 0.6	23 ± 2	29.5 ± 0.3	19.8 ± 0.4	27.9 ± 0.4
**D_{max} (Å)**	87-120	84-108	105 - 121	98 - 125	70 - 96	91-115
**q-RANGE (Å^{-1})**	0.016 - 0.30	0.016 - 0.33	0.046 - 0.50	0.015 - 0.28	0.015 - 0.42	0.018 - 0.27
**GNOM QUALITY**	0.79	0.76	0.52	0.72	0.80	0.69
**VOLUME THEORETIC**	60177.2					
**POROD VOLUME**	55500	43600	40600	76700	59300	51000
**Shape modeling results**						
**SYMMETRY ASSUMPTION**	P1					
χ^2 **MONSA**	5.3	5.6	3.8	9.7	8.0	1.5
**I(0) MONSA/I(0)$_{experimental}$**	0.93	0.93	0.33	1.0	0.94	1.0
**q-RANGE FOR FITTING (Å^{-1})**	0.018 - 0.25					

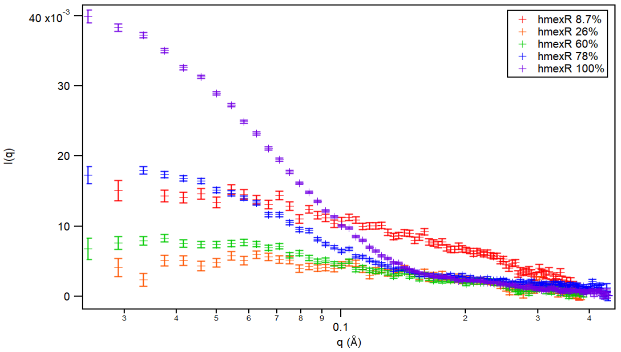

(a) Data set of hMexR.

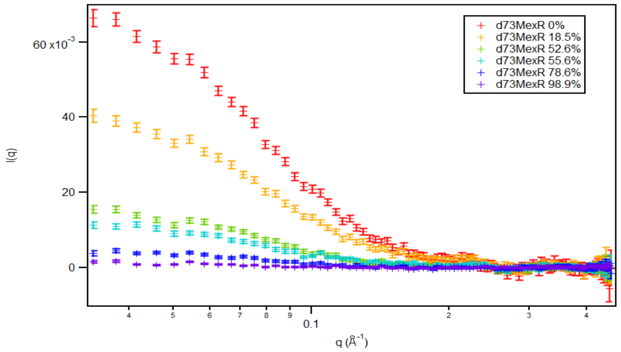

(b) Data set of d73MexR.

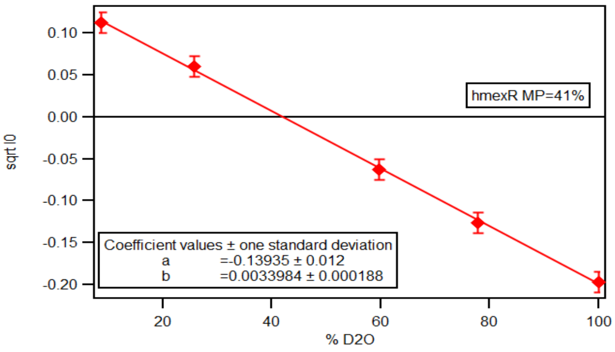

(c) Linear fit for the measurement of MP of hMexR.

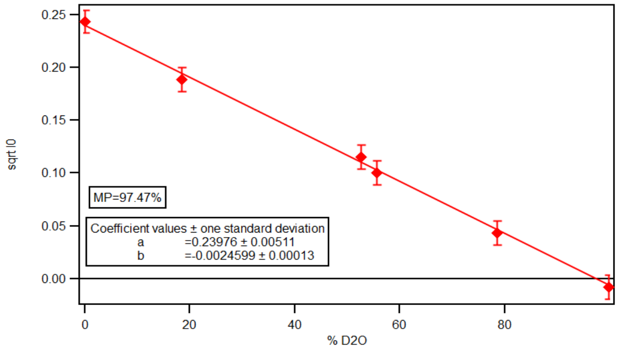

(d) Linear fit for the measurement of MP of d73MexR.

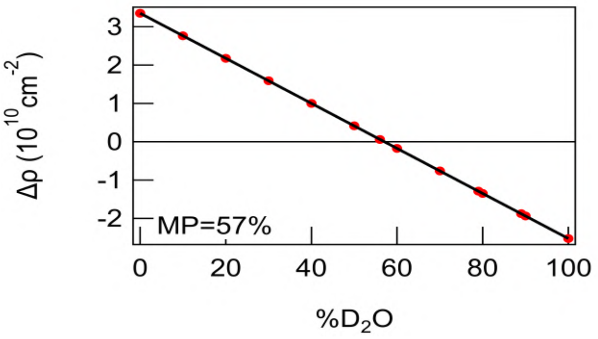

(e) Theorical MP for DNA

Figure S3: **Experimental determination of hMexR and d73MexR match points and theorical for PII DNA.** Panels a and b show the data sets of hMexR and d73mexR at different $\%D_2O$. We acquired the data at D22 with a 2 m detector distance setup, and all samples analysed in this experiment were at 6 mg ml^{-1}. For the measure of the MPs, we performed a linear fit over $\sqrt{I_0}$ vs $\%D_2O$. From the linear fit the MP are extract and they are respectively 41% and 97%. We reduced the data with GRASP software, and the analysis was performed using IGOR Pro v.7.

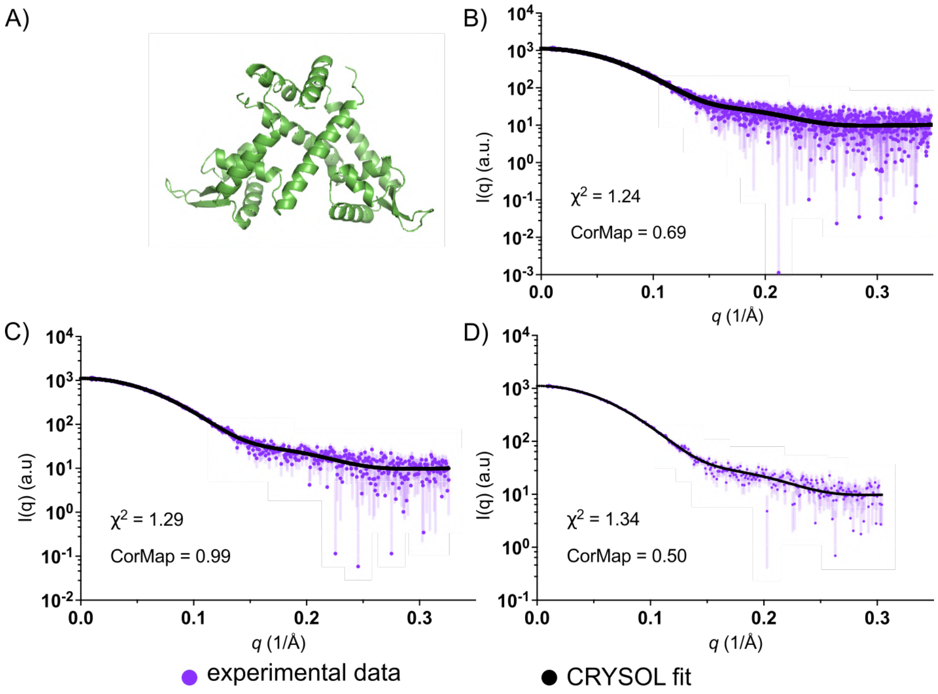

Figure S4: **Examples of re-binned data sets.** The errors are shown in light purple. A. sREFLEX refined model based on 1LNW PDB entry, amino acids Val5-Leu139. B. Scattering profile, presented as I(q) vs q collected at P12 BioSAXS beamline (EMBL-HH). The 2D-to-1D data reduction, including radial averaging, was performed using the SASFLOW pipeline incorporating RADAVER (ATSAS 2.8). 1249 data points. C. Re-binned data using DATREGRID, 624 data points D. Re-binned data using DATREGRID, 416 data points.

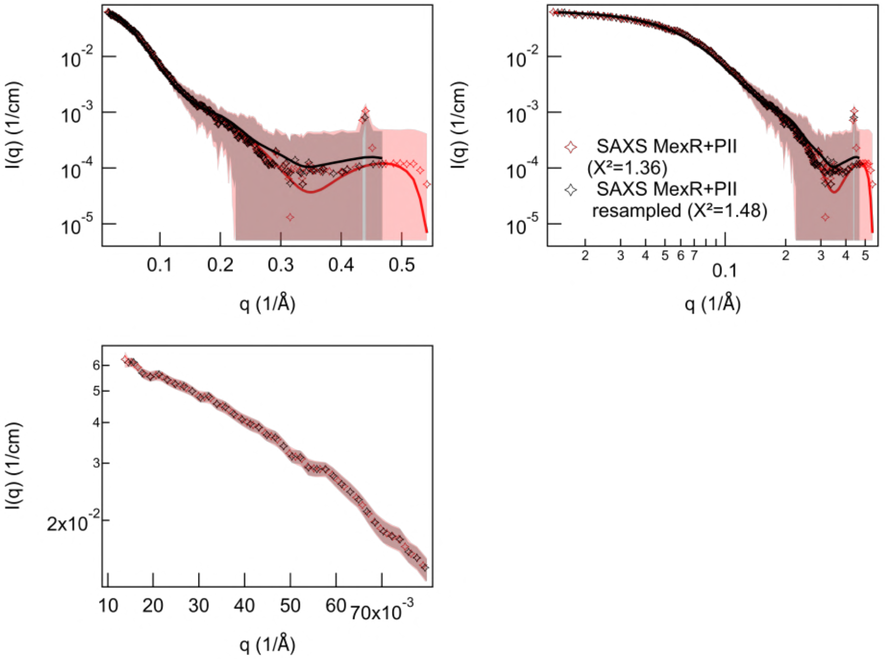

Figure S5: **Binning of SAXS data for MexR+PII.** The top panels show the data before (red) and after (black) the rebinning in both Log(I) vs log(q) and log(I) *vs.* q. The lines represent the results of the PEPSI-SAXS fits of the structure in Figure 3B on the original paper. The bottom panel shows a zoom at high q-values. In all figures, the error bars are represented as a semitransparent continuous band to help the visualisation of the point and the error bands.

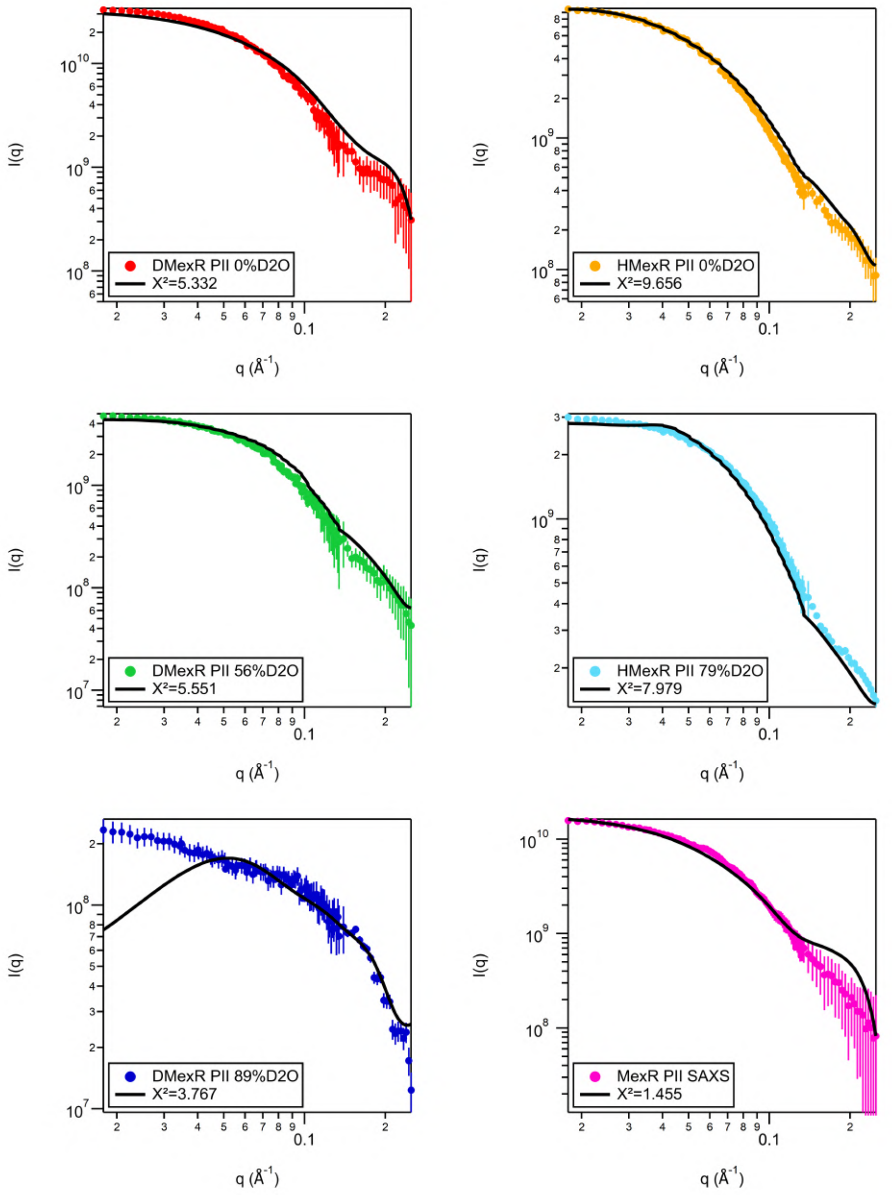

Figure S6: **SANS data set of MexR-PII complex supplemented with laboratory SAXS data of fully-protonated MexR-PII with MONSA fit.** The graphs show the reduced data of the experiment at D22 of the MexR bound with PII DNA. The black lines passing through the experimental point is the MONSA fit of the structure shown in figure 3B. Colorcode: SAXS of fully protonated complex (pink), SANS of fully protonated in 0% (yellow) and 79 % (cyan) D_2O buffers, and SANS of the dMexR-PII complex in 0 % (red), 56 % (green) and 89 % (navy) D_2O buffers. The shown data are the direct outcome from MONSA and they have a difference in intensity, because the output file of MONSA is the fit of the simulated scattering curve versus a smoothed-out version of the real-data multiply by a constant.

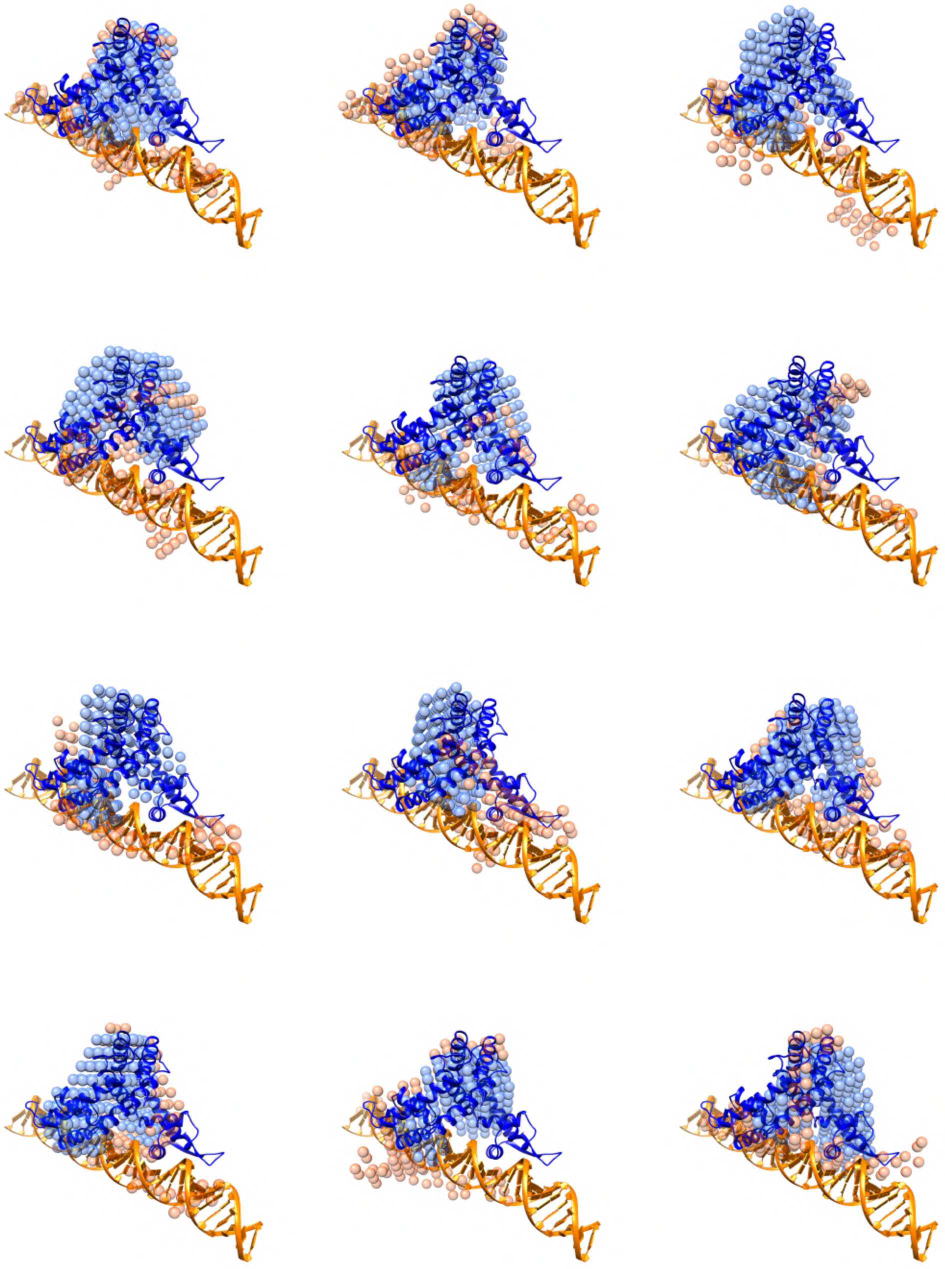

Figure S7: **MONSA for MexR-PII DNA.** Twelve results of MONSA *ab-initio* analysis performed to the MexR-PII data set.

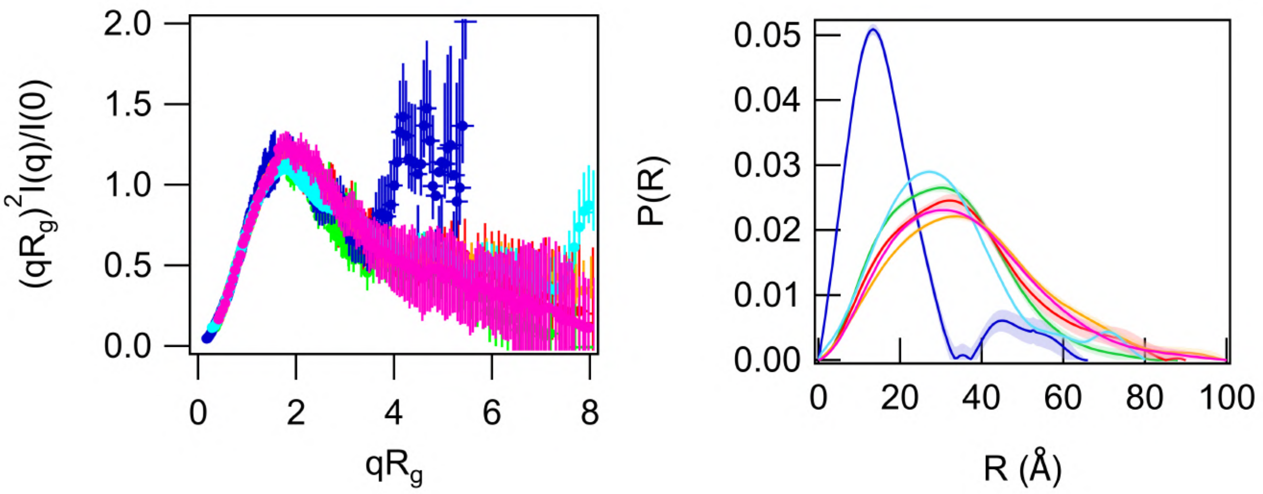

Figure S8: **Dimensionless Kratky Plot and *P(r)* MexR-PII.** The graphs show the Dimensionless Kratky plot of the data set of MexR-PII and distances of distribution of the data set acquired. The *P(r)* is calculated with the ATSAS software and normalised by the area, and the light area represents the uncertainty that ATSAS software give for the *P(r)*. Colorcode: SAXS of fully protonated complex (pink), SANS of fully protonated in 0% (yellow) and 79 % (cyan) D_2O buffers, and SANS of the dMexR-PII complex in 0 % (red), 56 % (green) and 89 % (navy) D_2O buffers. Note that dMexR-PII in 89% D_2O is not recorded at match point and therefore contains both the contribution from the DNA and the protein.

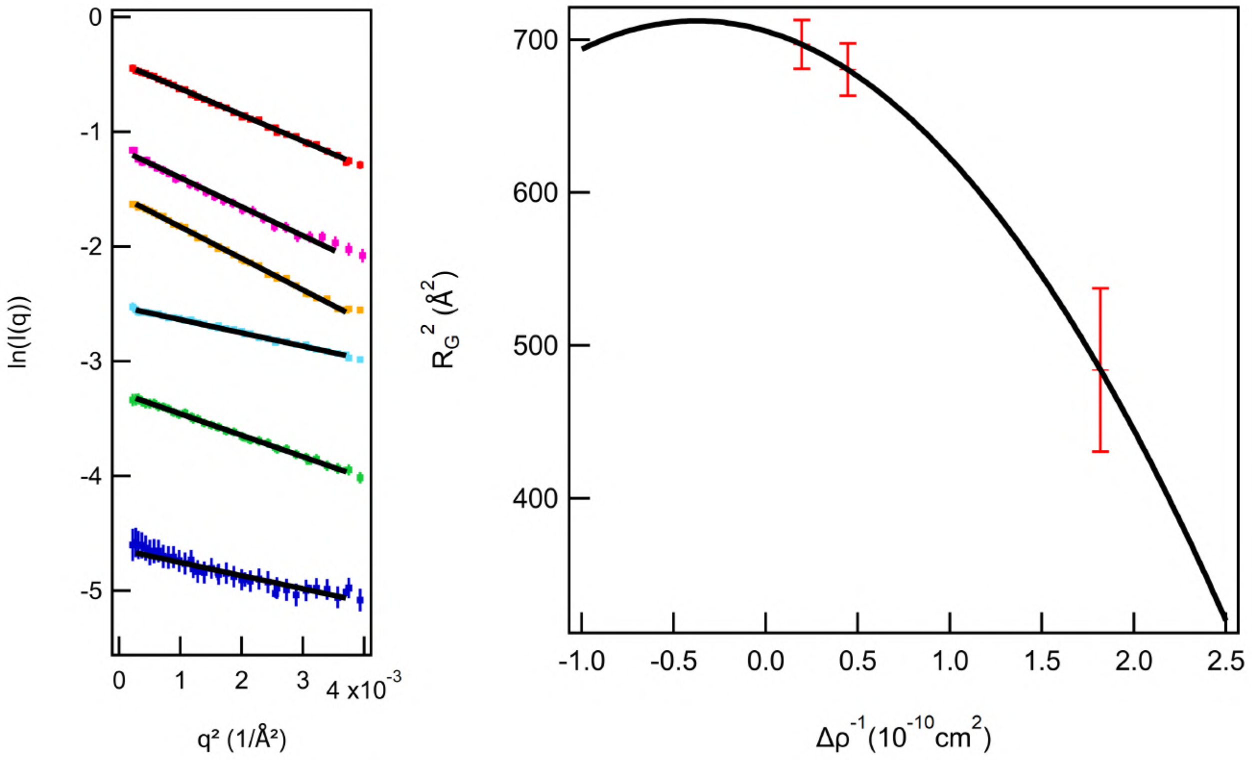

Figure S9: **Guinier fit for all the data set for MexR-DNA performed with the NCNR macro for IGOR and Stuhrmann plot.** Left plot: Color code: SAXS of fully protonated complex (pink), SANS of fully protonated in 0% (yellow) and 79 % (cyan) D_2O buffers, and SANS of the dMexR-PII complex in 0 % (red), 56 % (green) and 89 % (navy) D_2O buffers. The ranges and the results of the linear fits are shown in table S2. Right plot: Stuhrmann plot of the set of data with partially deuterated protein with its parabolic fit $R_g^2 = R_v^2 + \frac{\alpha}{\Delta\rho} - \frac{\beta}{\Delta\rho^2}$ used as a guide for the eyes. Since $\beta \neq 0$ this tell us that the components do not have the same center of mass, and since $\alpha < 0$ this indicates that the more dense component (*ie* the protein) is closer to the center of mass (40). Both of these conclusions agree with *ab-initio* and molecular models as presented in Figure 4B.

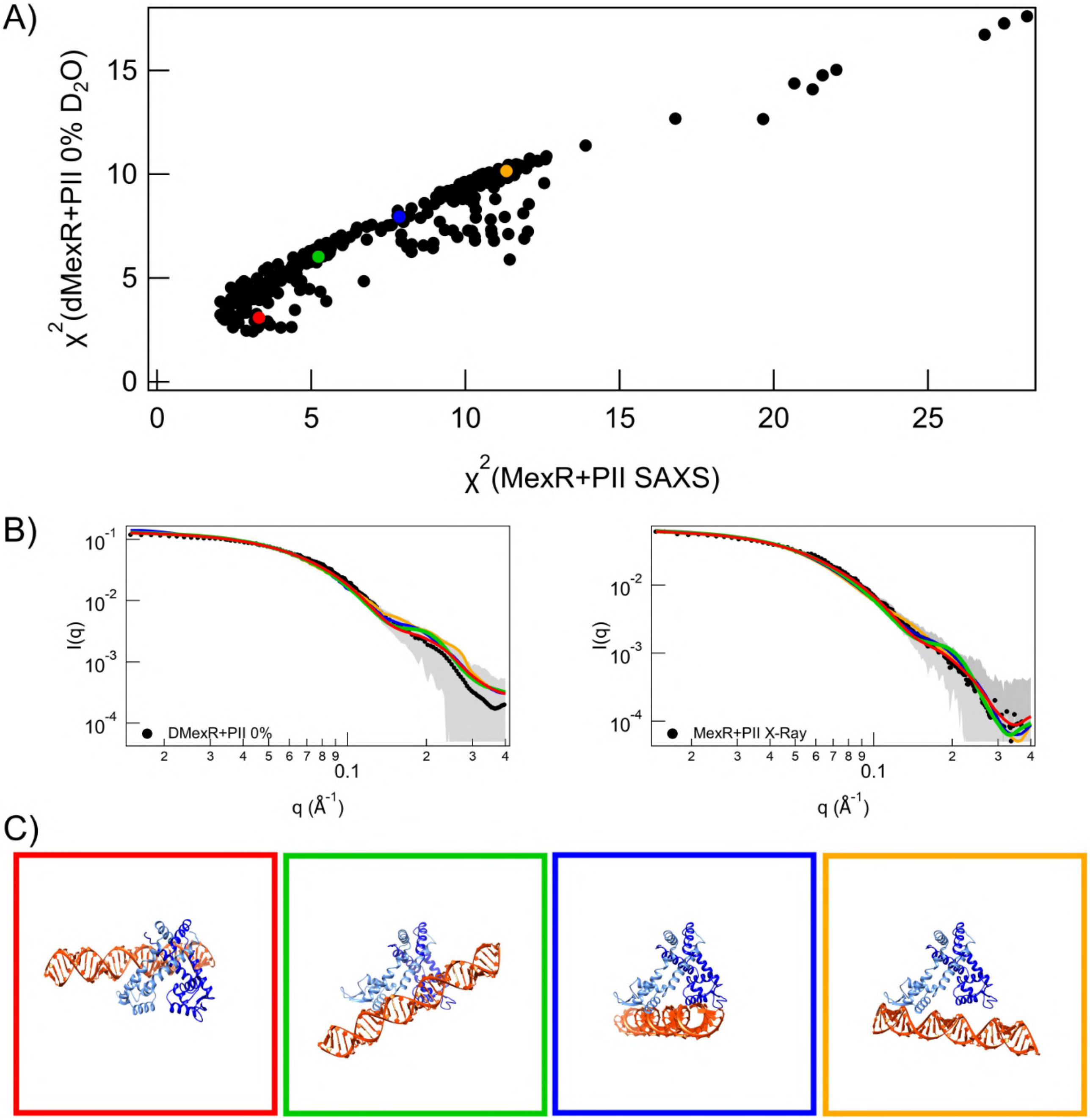

Figure S10: **H-Dock Docking results.** A) Scatter plot of the χ^2 of dmexR PII 0% D_2O over the χ^2 of MexR PII SAXS data. It is possible to notice a linear trend, but still the χ^2 are all above 2. B) The graphs represent the overlap between the experimental data (black dots) and the simulated curves. The structures were generated with the docking, and they are listed below in C). The colours of the curves correspond to the colours of the frames of the structures. C) Four examples of structures generated with H-Dock. For clarity, the orientation of the MexR is the same in all the pictures

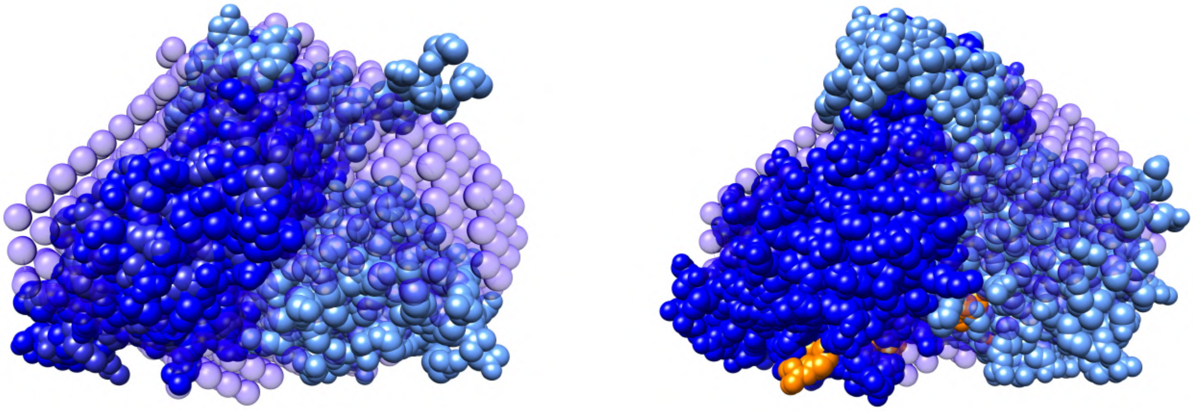

Figure S11: DAMFILT *ab-initio* model (beads) superposed with the 4 structures of 1LNW (left) and the representatives of the best-fitting ensemble of 21 structures representing apo-MexR shown in figure 4D) (right).

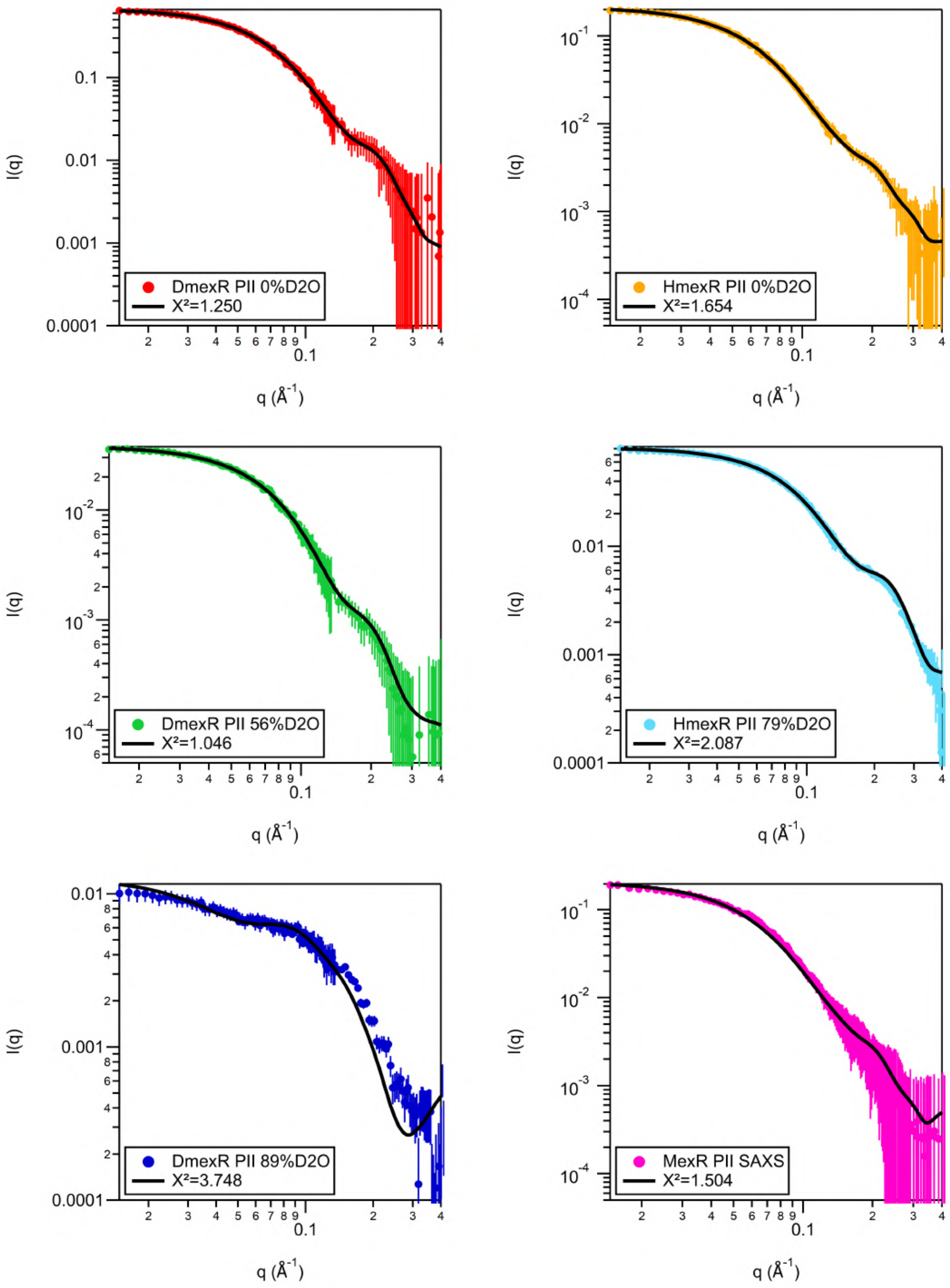

Figure S12: **SANS data set of MexR-PII complex supplemented with laboratory SAXS data of fully-protonated MexR-PII with CRYSOL/N fit.** The graphs show the reduced data of the experiment at D22 of the MexR bound with PII DNA. The black lines passing through the experimental point is the CRYSOL/N fit of the structure shown in figure 3B. Colorcode: SAXS of fully protonated complex (pink), SANS of fully protonated in 0% (yellow) and 79 % (cyan) D_2O buffers, and SANS of the dMexR-PII complex in 0 % (red), 56 % (green) and 89 % (navy) D_2O buffers. The experimental data are the same as in Figure S6 even they have discrepancy in intensity and q-range.
