# Supplementary material for: Small-angle x-ray and neutron scattering of MexR and its complex with DNA supports a conformational selection binding model
Source: Biophys J. 2022 Dec 5;122(2):408–18. doi: 10.1016/j.bpj.2022.11.2949 (PMC9892617; doi:10.1016/j.bpj.2022.11.2949)
Supplement: Document S2. Article plus supporting material [file mmc2.pdf]

Biophysical Journal
Article

Small-angle x-ray and neutron scattering of MexR and its complex with DNA supports a conformational selection binding model

Francesca Caporaletti,[1,2] Zuzanna Pietras,[1] Vivian Morad,[1] Lars-Göran Mårtensson,[1] Frank Gabel,[3] Björn Wallner,[1] Anne Martel,[2] and Maria Sunnerhagen[1,*]
[1]Department of Physics, Chemistry and Biology (IFM), Linköping University, Linköping, Sweden; [2]Large Scale Structure, Institute Laue Langevin, Grenoble, France; and [3]University Grenoble Alpes, CEA, CNRS, IBS, Grenoble, France

ABSTRACT In this work, we used small-angle x-ray and neutron scattering to reveal the shape of the protein-DNA complex of the *Pseudomonas aeruginosa* transcriptional regulator MexR, a member of the multiple antibiotics resistance regulator (MarR) family, when bound to one of its native DNA binding sites. Several MarR-like proteins, including MexR, repress the expression of efflux pump proteins by binding to DNA on regulatory sites overlapping with promoter regions. When expressed, efflux proteins self-assemble to form multiprotein complexes and actively expel highly toxic compounds out of the host organism. The mutational pressure on efflux-regulating MarR family proteins is high since deficient DNA binding leads to constitutive expression of efflux pumps and thereby supports acquired multidrug resistance. Understanding the functional outcome of such mutations and their effects on DNA binding has been hampered by the scarcity of structural and dynamic characterization of both free and DNA-bound MarR proteins. Here, we show how combined neutron and x-ray small-angle scattering of both states in solution support a conformational selection model that enhances MexR asymmetry in binding to one of its promoter-overlapping DNA binding sites.

SIGNIFICANCE Several MarR-like proteins regulate the expression of efflux pumps, actively expelling highly toxic compounds out of the host organism. Antibiotics resistance mutations lead to continuous production of efflux proteins and increased bacterial survival. MexR is a MarR family member in the pathogen *Pseudomonas aeruginosa*, where mechanisms for its selective DNA binding remain unclear. In this work, we used small-angle neutron and x-ray scattering to evaluate the shapes of MexR in solution, free, and bound to its native DNA target. We find direct evidence of a DNA-binding conformational selection mechanism, where MexR conformations equivalent to the bound state are present already in the absence of DNA. Our work helps understanding how antibiotics resistance is regulated and how it could be defeated.

INTRODUCTION

The regulatory interactions of transcription factors represent one of the most dynamic biological response systems in the cell (1). Transcription factors interact both with DNA and other proteins involved in regulatory complexes and need to respond swiftly and accurately to changes in cellular *stimuli*. Over the past four decades, fundamental achievements have been made in the structural understanding of protein-DNA binding ranging from complexes of DNA with single protein monomer and dimers to multiprotein complexes, entire transcription factor assemblies, and even nucleosomal particles (2). The helix-turn-helix (HTH) DNA-binding motif is extensively used by transcriptional regulators in both prokaryotes and eukaryotes, enabling efficient and versatile DNA binding (3). In the winged-HTH motifs (wHTH), an additional β-finger extends the DNA contact surface beyond the major groove-HTH contacts (4). The wHTH superclass contains the majority of prokaryotic transcription factors, including the multiple antibiotics resistance regulator (MarR) family to which MexR belongs (3).

Pseudomonas aeruginosa is a Gram-negative bacterium and an opportunistic pathogen that can cause severe nosocomial infections (5). As a consequence of its inherent antibiotic resistance and its pathogenic potential, together with increasing concern of infection of immunocompromised

Submitted March 9, 2022, and accepted for publication November 29, 2022.
*Correspondence: maria.sunnerhagen@liu.se
Editor: Tommy Nylander.
https://doi.org/10.1016/j.bpj.2022.11.2949
© 2022 Biophysical Society.
This is an open access article under the CC BY license (http://creativecommons.org/licenses/by/4.0/).

patients in hospitals, it has been described as a "priority pathogen" by the World Health Organization (6). Inherent multidrug resistance in *P. aeruginosa* is mainly attributable to an interplay of low outer membrane permeability and increased expression of protein-forming efflux pumps, which recognize substances toxic to the bacteria, including multiple clinical antibiotics such as quinolones, β-lactams, tetracycline, chloramphenicol, and novobiocin, and expel them from the cell (7,8). Incapacitating the transcriptional repressor protein leads to constitutively high production of the efflux proteins and thus increased survival for the bacteria (8). The mechanism is common to a wide range of bacteria, and members of the MarR family of proteins are ubiquitous in both archaea and bacteria (9,10).

The MexR repressor inhibits gene expression by binding to two DNA regions, designated PI and PII, which overlap with promoter regions of MexR as well as the MexA-MexB-OprM efflux operon, comprising genes of the MexAB-OprM efflux pump (Fig. 1 *A*) (11,12). The PI and PII sites each contain pairs of inverted palindromic GTTGA sequences, suggesting one MexR dimer binding at each PI and PII site (11), but outside the palindromes the PI and PII sites are highly asymmetric (Fig. 1 *A*). As a member of the homodimeric MarR family of wHTH transcriptional regulators, the MexR homodimer holds a triangular shape and pseudo-two-fold symmetry, where amino- and carboxy-terminal helices interdigitate to create a dimerization interface that supports DNA binding by two helix-turn-helix motifs (13) (Fig. 1 *B*). The Cα distance between conserved DNA-anchoring arginines in each α4 helix (Arg73/73′ in MexR) has been used as an indicator of DNA binding suitability in the MarR family, as it reflects the distance between the two recognition helices and can been used as an indicator of DNA binding suitability ((8,13); see Fig. 1 *B*).

No high-resolution structure of MexR bound to DNA has been obtained yet, possibly due to structural variability. The first crystal analysis of MexR in the absence of DNA showed four distinct dimer conformations, jointly suggesting a two-state open-closed model, where an inhibitor-induced conformational change would reduce the distance between the α4 helices and thereby disable DNA binding (13). In the MarR family protein OhrR, consecutive structural displacements were proposed to enable transition between apo- and DNA-bound states (14). Antirepression would then interrupt this path, as in the crystal structure of the MexR dimer complexed with the antirepressor protein ArmR (15). The regulation of DNA binding also responds to stress-induced cellular changes, which promote cysteine-cross-linking inactivating both MarR and MexR (16), thereby abolishing DNA binding (17). In our previous work (18), we showed that molecular dynamics simulations suggest that MexR exists in a wide range of conformations, including a subpopulation similar to the DNA-bound conformation of the MexR homolog OhrR (14), which we then used as a template for the MexR-DNA bound state. Furthermore, access to the DNA-bound state within the MexR-apo ensemble was limited by a mutation distant from the DNA binding site restraining the conformational ensemble, suggesting conformational selection as the prevalent DNA-binding mechanism for MexR (18).

To further investigate mechanisms for MexR-DNA binding in solution, and to provide more information on MexR-DNA bound state(s), we here provide a comprehensive small-angle scattering (SAS) analysis of the free MexR dimer as well as of the MexR-DNA complex. Contrast variation SAS data jointly with forward molecular modeling show that the ensemble of DNA-bound MexR conformations identified by Small Angle Neutron Scattering (SANS) is largely a subensemble of the larger apo-MexR ensemble, supporting conformational selection, and it structurally resolves the MexR interaction with its PII DNA binding site in solution, resulting in a molecular model that is structurally related to OhrR- and SlyA-DNA complexes (19) but with increased asymmetry. Jointly, our findings support a conformational selection mechanism for MexR-DNA binding that results in increased protein asymmetry in complex with a native, nonsymmetric DNA-binding site.

MATERIALS AND METHODS

Protein preparation

The DNA sequence encoding the MexR (Val5–Leu139, UniProt: P52003) was subcloned into the pNH-TrxT vector (Addgene plasmid 26106; http://n2t.net/addgene:26106; RRID:Addgene_26106 (20)), with a 6x-His-thioredoxin tag after a tobacco etch virus (TEV) protease cleavage site. Protein was overexpressed in *Escherichia coli* BL21 Rosetta 2(DE3), and bacteria were grown in Luria Broth medium at 37°C, in the presence of kanamycin (50 μ g/mL) and chloramphenicol (34 μ g/mL). Cultures were induced with 0.5 mM isopropyl β-D-1-thiogalactopyranoside (IPTG) at $OD_{600} = 0.8$, incubated overnight at 18°C, harvested by centrifugation, and lysed by sonication in lysis buffer (300 mM NaCl, 50 mM sodium phosphate buffer (pH 7.0), 10 mM imidazole, 10 mM β-mercaptoethanol (β-ME), 5% glycerol, 5 U/mL DNase I, and 1 $\times$ complete, EDTA-free Protease Inhibitor Cocktail (Roche Diagnostics)). The protein was purified by immobilized metal affinity chromatography (IMAC) using Ni-NTA-agarose resins at 4°C. The His-Trx tag was cleaved off with TEV protease, followed by a reverse IMAC purification step. The cleaved protein was purified on a HiLoad 16/600 Superdex 75 column (GE Healthcare) equilibrated in buffer containing 50 mM 4-(2-hydroxyethyl)-1-piperazineethanesulfonic acid (HEPES) (pH 7.0), 150 mM NaCl, 10% v/v glycerol, 1 mM tris(2-carboxyethyl)phosphine (TCEP) (20).

Partially deuterated (73% nonexchangeable ^{2}H) MexR was expressed using 100% D_2O M9 minimal medium (6 g/L sodium phosphate buffer, 3 g/L KH_2PO_4, 0.5 g/L NaCl, 0.1 mM $CaCl_2$, 1 mM $MgSO_4$, 10 mg/L biotin, 1 mg/L thiamine, 2 g NH_4Cl) with unlabeled glucose as a carbon source and purified as above (21,22).

Purified proteins were 95% pure as judged by SDS-PAGE. Molecular mass and corresponding degree of deuteration was obtained from matrix-assisted laser desorption/ionization-time of flight (MALDI-TOF) analyses (Bruker Daltonics). Protein concentration was determined by a Bradford assay (23) calibrated against the MexR-R21W mutant (18) due to the absence of tryptophan in wild-type MexR. Purified protein was concentrated using Amicon Centrifugal Filter (Millipore, regenerated cellulose membrane), flash-frozen in liquid nitrogen and stored at −80 ° C until further use.

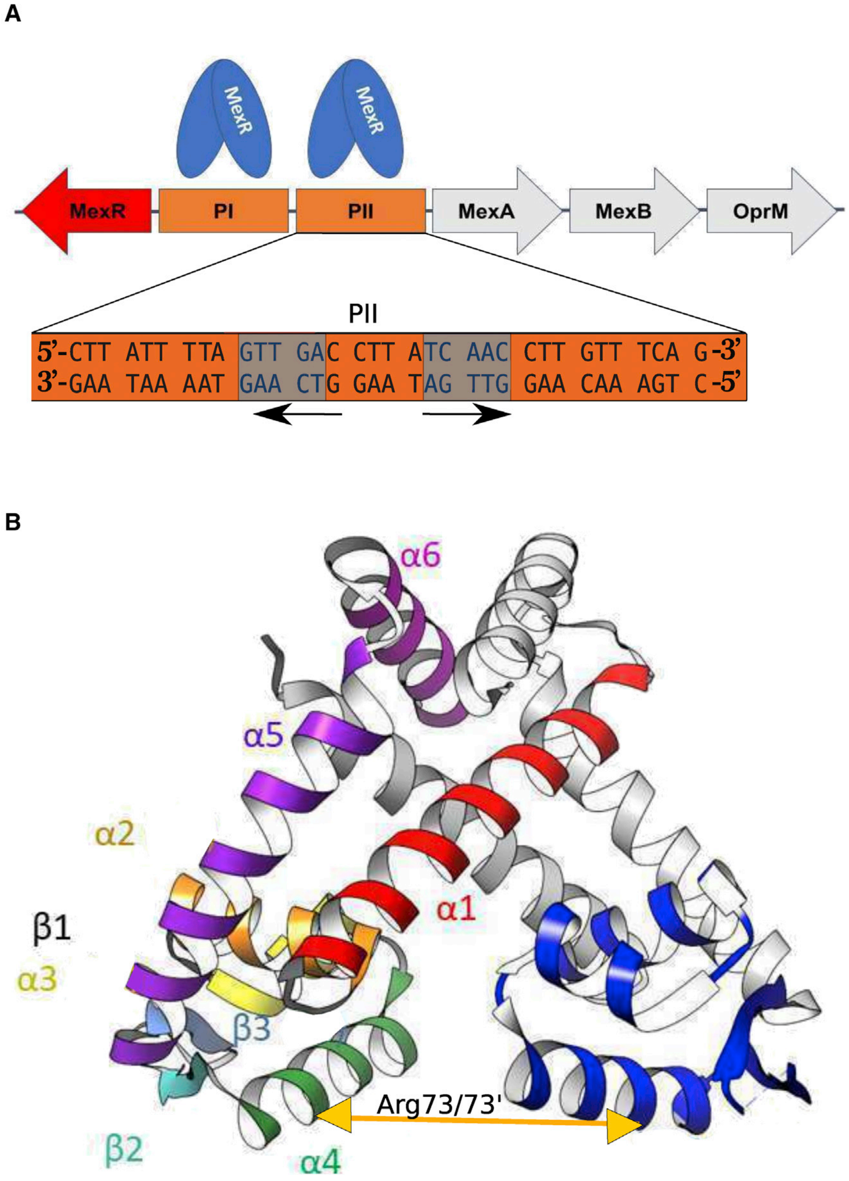

FIGURE 1 Overview of MexR structure and DNA-binding properties and MexR dimer. (*A*) Schematic representation of MexAB-OprM multidrug efflux operon; arrows indicate the direction of transcription. Location of MexR regulatory DNA binding sites PI and PII in orange, PII sequence detailed with palindromes in gray, and direction in black arrows. (*B*) Crystal structure of a representative MexR homodimer in the absence of DNA in ribbon representation (1LNW:CD). Each secondary structure element is individually labeled and colored on the left monomer. The winged-HTH motif is colored in blue in the monomer on the right and comprises α 2 β 1 α 3 α 4 β 2 (recognition helix) W1 (wing)- β 3. The distance between the two recognition helices (α 4/α 4′) is commonly reflected by the distance between Arg73-C_α in the two monomers (indicated by *yellow arrow*). To see this figure in color, go online.

DNA preparation

The DNA duplex was prepared from complementary 34-bp oligonucleotides (Eurogentech) corresponding to the PII MexR binding site within the PII_{MexA} promoter (11,12) (palindromes in boldface): forward: 5′- CTT ATT TTA **GTT GA**C CTT A**TC AAC** CTT GTT TCA G; reverse 5' - C TGA AAC AAG **GTT GA**T AAG G**TC AAC** TAA AAT AAG. The oligonucleotides were annealed in 5 mM β-mercaptoethanol, 20 mM sodium phosphate buffer, 150 mM NaCl (pH 7.0) at 95°C for 5 min, then allowed to cool down slowly to 30°C for 2 h. The resulting duplex was stored at 4°C or −20°C for longer storage. The buffer ensuring sample monodispersity is 150 mM NaCl, 20 mM sodium phosphate buffer (pH 7.1) and 10 mM DTT.

Isothermal titration calorimetry measurements

Isothermal titration calorimetry (ITC) experiments were performed on a MicroCal PEAQ-ITC instrument (Malvern). Before the experiment, MexR and dsDNA were dialyzed against 1 L of 5 mM β-mercaptoethanol, 20 mM sodium phosphate buffer, 150 mM NaCl at pH 7.1 and centrifuged at 3500 rpm for 10 min at the titration temperature (20 ° C). MexR at 105 μ M concentration was titrated with the initial injection of 0.4 μ L followed by 18 injections of 2.3 μ L into the 2 μ M dsDNA solution in cell. Data analysis was performed with MicroCal PEAQ-ITC Analysis Software (Malvern).

Size exclusion chromatography coupled to multiangle laser light scattering (SEC-MALLS)

The MALLS experiment was performed following SEC by in-line measurements using a Wyatt Technologies Mini-Dawn TREOS multiangle light scattering detector coupled to an OptiLab T-Rex refractometer (RI). Samples were injected onto Superdex 75 Increase 5/150 analytical column (GE Healthcare) equilibrated in 20 mM HEPES (pH 7.1), 150 mM NaCl, 10 mM DTT, 1% v/v glycerol, at a flow rate of 0.3 mL/min. The MALLS system was used at the incident wavelength of 659 nm, and in

	χ^2 PEPSI	χ^2 CRYSOL/N	χ^2 MONSA
DMexR-PII 0%	1.3	1.3	5.3
DMexR-PII 56%	1.0	1.0	5.6
DMexR-PII 89%	2.5	3.7	3.8
HMexR-PII 0%	1.7	1.7	9.7
HMexR-PII 79%	1.8	2.1	8.0
MexR-PII x-ray	1.5	1.8	1.5

combination with concentration estimates obtained from the RI (dn/dc = 0.185 mL/g) was used to evaluate the molecular weight (MW). The measurements were done at 20°C. The MW distribution of species eluting from the column was determined using ASTRA7 software (Wyatt Technology).

SEC-SAXS measurements of apo-MexR

SEC-SAXS scattering intensities *I(q)* collection of MexR was performed at 20°C at EMBL-P12-bioSAXS beamline (PETRAIII, DESY, Hamburg, Germany) (24). The scattering intensities *I(q)* versus q, where $q = 4\pi \sin(\theta)/\lambda$ with the x-ray wavelength $\lambda = 1.24$ Å (10 keV) and 2θ is the scattering angle. Column and elution parameters were the same as for the SEC-MALLS. The 35 μ L of sample at 8.5 mg/mL was injected. Only those SAXS data frames with a consistent radius of gyration (R_g) through the SEC elution peak and evaluated as statistically similar through the measured q-range (0.0024–0.73 Å $^{-1}$) were used to generate the final SAXS profile of MexR in solution using CHROMIXS software (25). The experiment and data evaluation are further detailed in Table S1. The tables in the supplementary are inspired by the guidelines described in Trewhella et al. (26). The comparison between theoretical scattering calculated from molecular structures and experimental data was performed with PEPSI-SAXS (27); the goodness-of-fit was estimated by evaluating the lowest χ^2 between model and experimental scattering curves and was not dependent on the binning of data (Fig. S4). Experimental errors were in agreement with what is expected for protein solutions at the studied MW and at the concentrations used.

Batch SAS measurements of the MexR-DNA complex

SANS experiments of the MexR-DNA complex were conducted on the D22 instrument at the Institut Laue Langevin (ILL), Grenoble, France, with an incident wavelength $\lambda = (6 \pm 0.6)$ Å. For SANS contrast variation series, the hydrogenated (hMexR) or deuterated (dMexR) MexR were mixed with equimolar DNA duplex, and the complex was dialyzed against buffer containing 20 mM sodium phosphate buffer (pH 7.1), 150 mM NaCl, 10 mM DTT with D_2O concentration of 0 and 79 v/v % for hMexR-PII and 0, 56, 89 v/v % for dMexR-PII. The 6.2 mg/mL samples were measured at 10°C in rectangular Hellma cuvettes of 1 mm thickness. Two different sample-to-detector distance/collimator setups were used to cover the q-range of $0.014 - 0.5$ Å^{-1}: 5.6/5.6 and 1.4/2.8 m. Data acquisition and instrument control were done using NOMAD software (28). The raw scattering patterns were processed using GRASP software (29), which includes azimuthal averaging, blocked-beam and empty cell subtraction, transmission, thickness, and monitoring count normalization and scaling to absolute intensity (*I(q)*, cm^{-1}) using direct flux measurement and water normalization. Good statistics are strongly dependent on the instrument, the detector, and the data collection. A certain number of neutrons N_t on the whole detector will after radial averaging give good statistics (30). The experiment times were optimized to gain as much signal over time as possible given the concentration and molecular mass of the protein complex that means a difference $\Delta N_t = 1,000,000$ from the sample over the buffer. A short acquisition of 10 s was made for each setup to estimate the sample count rate c/s, where the desired $\Delta(N_t)/\Delta(c/s)$ gives the acquisition time (see Table S2), where $\Delta(c/s)$ is the difference between the sample and the buffer count rate, with the acquisition time for the buffer kept the same as the sample. Further corrections of the experimental noise (31) were not required since subsequent data evaluation included fits to data with the same number of degrees of freedom (32). Curve merging, buffer subtraction, and Guinier fit that is defined as $\ln I(q)$ versus q^2, for $qR_g < 1.3$ (33) was performed using IGORpro SANS-reduction NCNR macros (34).

SAXS data of the MexR-DNA complex were acquired using Anton Paar SAXSess in slit geometry at Linköping University, operated at a wavelength of 1.54 Å (CuK α wavelength) coupled with a CCD camera. Data were placed on an absolute scale using the known scattering from pure water and reduced to $I(q)$ versus q for the protein by subtraction of the solvent blank. The data reduction was performed with the proprietary software SAXSquant (Anton Paar), including background subtraction, absorption correction, and desmearing correction, which is based on the Lake algorithm (35). The 3.12 mg/mL sample and corresponding buffer were measured at 10°C in a capillary of 1 mm thickness. Experiment parameters and their evaluation are detailed in Table S2. Binning of SAXS data as highlighted in Fig. S5 was done to match the SANS data points in subsequent SAS-based modeling and did not affect the χ^2, as shown in Fig. S5. Experimental errors at the SAXSess in slit geometry were in agreement with what is expected for protein solutions at comparable MWs and concentrations (36).

SAS data processing and modeling

SAS data analysis was performed using programs from the ATSAS package (37). Structural parameters were derived using Guinier analysis and from the inverse Fourier transformation method. The pair distance distribution *P(r)* plots of experimental data were determined using GNOM (38) from which R_g, D_{Max} (the estimated maximum particle dimension), and $I(0)$ (the extrapolated intensity at zero angles) values were estimated. Both the neutrons and x-ray scattering length densities (SLDs) were calculated using MULCh (39). Data acquisition and evaluation are further detailed in Table S1 for apo-MexR and Table S2 for the MexR-PII complex. The Guinier fit within the Stuhrmann plot (40) of the MexR-PII complex is shown in Fig. S9.

The program DAMMIF (41) was used for ab initio low-resolution shape reconstruction of apo-MexR; as a target function, it uses the reciprocal-space fit of the *P(r)* calculated from SAXS data and constrained to zero at $q = 0$ and $q = D_{Max}$. Ten independent ab initio-models of apo-MexR were generated using DAMMIF, and the individual models were spatially aligned using the DAMAVER package, including DAMSEL and DAMSUP (42), to generate a spatial representation of the protein, i.e., a final low-resolution model, taking into account the particle volume and consistent structural features across the model cohort (DAMFILT; (43)).

As a basis for the molecular modeling of the MexR-DNA complex, an ensemble of previously generated MexR structures by MD simulations were used (18). In short, four MexR-wt crystal structures from Protein Data Bank (PDB: 1LNW, chains CD, EF, and GH, selecting MexR residues 5–139 for all proteins) were used as starting structures in MD simulations using Gromacs 4.5.5 (44) for each of the four different couple of chains, totaling in 4 μs ($10 \times 4 \times 100$ ns) simulation time, generating an ensemble of 15,000 MexR conformations. All comparisons between theorical scattering calculated from molecular structures of the MD simulations were performed with hydrogenated protein in H_2O. Protein-DNA interactions were explored using HDOCK (45) with default settings and with the upload with the structures generated with MD simulations and with minimum χ^2, for the molecule and the DNA ligand. Template-based docking using OhrR protein-DNA complex (PDB ID: 1Z9C) was used to generate a structure of MexR bound to DNA. First, PII B-DNA was aligned to the DNA of MarR proteins OhrR and SlyA, using the dyad center as a match point (MP). Selected MexR models of the dimer with lowest χ^2 were then aligned to OhrR and SlyA dimer, placing the MexR dimer in an approximate

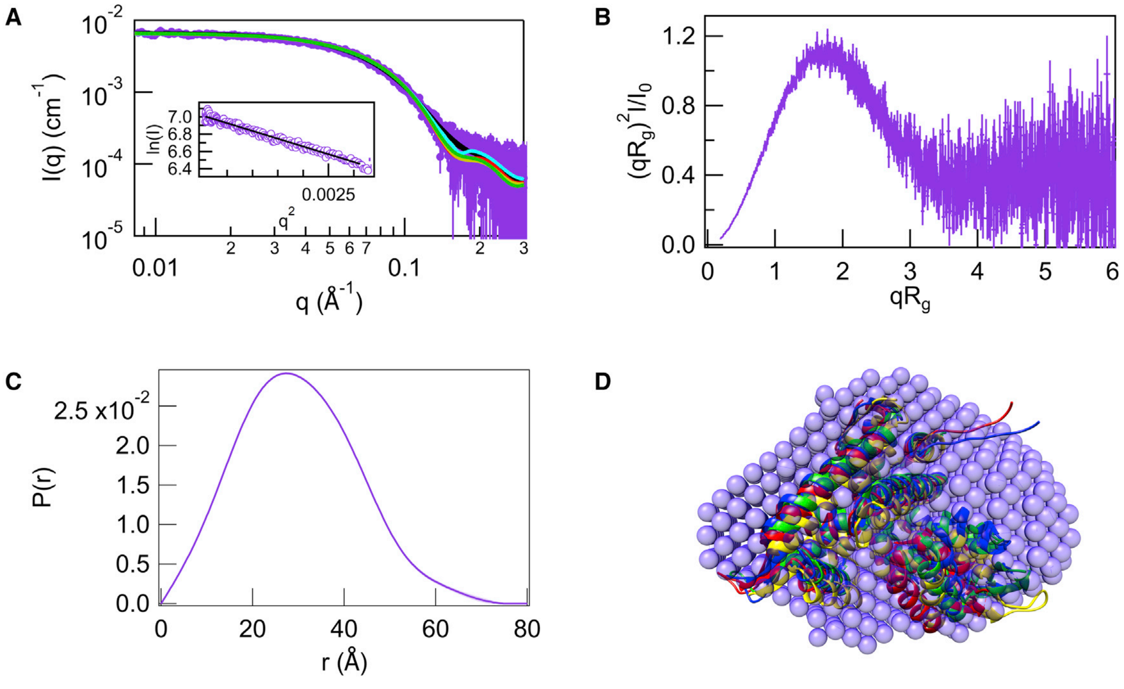

FIGURE 2 SEC-SAXS results for apo-MexR. (*A*) The final averaged SAXS profile of MexR thought SEC elution peak. In purple are the experimental data and in black the DAMFILT curve obtained from the DAMFILT volume shown in (*D*) ($\chi^2 = 1.30$); curves show the best fit of the theoretical scattering curves of MexR structures 1LNW:AB (*red*, $\chi^2 = 1.60$), 1LNW:CD (*blue*, $\chi^2 = 1.41$); 1LNW:EF (*yellow*, $\chi^2 = 1.58$), and 1LNW:GH (*green*, $\chi^2 = 1.47$). Insert: the corresponding Guinier plot and linear fit, where the R_g was assed at 23.2 Å within the sR_g limit of $0.2 < sR_g < 1.3$. (*B*) Dimensionless Kratky plot of the SAXS profile of apo-MexR. (*C*) *P(r)* versus *r* profile calculated from the SAXS data. (*D*) DAMFILT ab initio model (beads) superposed with 1LNW:AB, CD, EF, and GH structures in ribbon representation, colored as in (*A*). To see this figure in color, go online.

DNA-bound location. The MexR-DNA complexes were then energy minimized using the relax protocol in Rosetta (46).

Multiphase ab initio shape reconstructions were used to describe the MexR-PII complex (47,48). To distinguish MexR and DNA positions within the complex, SANS and SAXS curves were simultaneously used for multiphase ab initio reconstruction using MONSA software (48). SLD and volume fraction of each phase was estimated using the MULCh (39). The inputs for the MONSA analysis are summarized in Table S2. The comparison between theoretical scattering curves from molecular models and experimental scattering curves was performed using PEPSI-SAXS/SANS (27), and the goodness-of-fit was estimated by evaluating the lowest χ^2 between theoretical and models and experimental scattering curves.

To estimate the asymmetry of the MexR, we used a variant of a measure previously used to measure symmetry (49). Let A and B be any two atoms in one chain of the dimer and A′ and B′ be the corresponding atoms in the other chain. Then a perfectly symmetric dimer would have $dist(\mathrm{A}, \mathrm{B}') = dist(\mathrm{A}', \mathrm{B})$ for all pairs of atoms. Asymmetry is defined as the average of $|dist(\mathrm{A}, \mathrm{B}') - dist(\mathrm{A}', \mathrm{B})|$ over all pairs of C α. This measure will deviate from zero when the MexR is asymmetric.

RESULTS

MexR binding to PII DNA

In this study we investigated the MexR interaction with native, nonsymmetrized PII DNA (Fig. 1 *A*). The binding affinity of MexR to PII dsDNA was measured using ITC. MexR binds dsDNA with a K_D of 240 nM (Fig. S1). This is comparable to previous measurements by surface plasmon resonance ($K_D = 370$ nM) (12). These affinities are in good-agreement with previously observed MarR affinities to nonsymmetrized promoters (50–53). The stoichiometry of MexR binding to PII obtained by ITC implies the binding of one homodimer of MexR to a single PII duplex DNA. The thermodynamic profile suggests an entropically driven complex formation, in agreement with other MarR proteins (50).

SEC-SAXS evaluation of the apo-MexR dimer

The SEC-MALLS-RI trace of the MexR protein sample and the corresponding protein MW correlation calculated through the major elution peak are shown in Fig. S2. The MW was calculated from the static light scattering intensities and protein concentration estimates. The MW of MexR is within the range of 34–38 kDa, with an MW average of 35.5 kDa. The expected MW of MexR calculated from the amino acid sequence is 15.3 kDa. Therefore, the MexR elutes from the SEC column as a dimer.

Synchrotron SEC-SAXS measurement provided high-quality data of MexR in the absence of DNA, devoid of any obvious traces of aggregation (Fig. 2 *A*) (Table S1). The calculated R_g is slightly lower (23.2 Å; Fig. 2 *A*) than the one extracted from previous data recorded without SEC (25.8 Å) (18). This difference could be due to SEC removal of slight MexR aggregation in the absence of

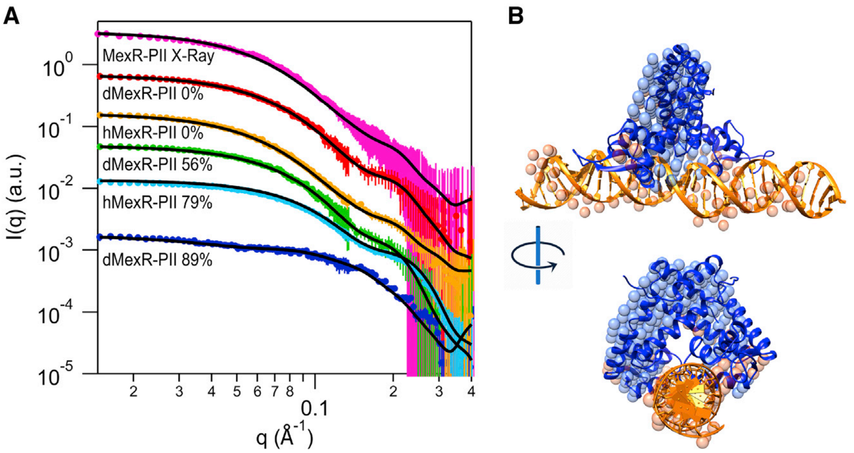

FIGURE 3 Characterization of MexR-PII complex by small-angle scattering. (*A*) Scattering curves registered at different contrast: SAXS of fully protonated complex (*pink*), SANS of fully protonated in 0% (*yellow*) and 79% (*cyan*) D_2O buffers, and SANS of the dMexR-PII complex in 0% (*red*), 56% (*green*), and 89% (*navy*) D_2O buffers. Continuous black lines show the best fit of the atomic model (see results section SAS-based molecular modeling of MexR and the MexR-DNA complex). SAS curves and fits have been scaled for better visibility. MONSA fits to data are shown in Fig. S6. (*B*) Beads model showing the ab initio reconstructed MexR (*blue*)-DNA (*orange*) complex, with molecular model superimposed (*in ribbon*, same color scheme). Table insert shows corresponding χ^2 fits-to-data for the molecular model (PEPSI), χ^2 fits-to-data for the molecular model (CRYSOL/N, in Fig. S12, there are the corresponding curves), and the ab initio reconstructed model (MONSA). To see this figure in color, go online.

DNA and/or to further optimization of construct design and buffer conditions (see materials and methods for details). A dimensionless Kratky plot representation of the SAXS data is shown in Fig. 2 *B*. A symmetric "bell-shaped" peak is observed in the dimensionless Kratky plot, suggesting that MexR likely adopts an overall compact/globular conformation in solution. *P(r)* analysis agrees on a compactly folded dimer unit with maximum dimensions of 77 Å (Fig. 2 *C*). Overall, the *P(r)* profile displays a reasonable symmetric distribution of distances. In agreement, the overall shape of the DAMMIF envelope for MexR is consistent with a compact triangular structure characteristic of MarR family (Fig. 2 *D*). Previous crystal structure conformations for the MexR dimer (13) all fit with similar χ^2 to the SAXS data, supporting that the ensemble of conformations observed in the crystal is also present in solution (18). Taken together, SAXS data thus show that MexR is a dimer in solution, and ITC data show that it also binds DNA as a dimer. We will therefore refer to the MexR dimer as "MexR" from now on.

SAS-based ab initio evaluation of the MexR-DNA complex

In order to experimentally resolve the DNA-bound complex of MexR with the PII DNA, we combined SAXS and SANS with contrast variation data. Two protein-DNA complexes were produced: one with MexR deuterated to 73% of nonexchangeable hydrogens and protonated DNA (dMexR-DNA), and the second complex where both the DNA and the MexR were fully protonated (hMexR-DNA). In Fig. 3 *A* all the recorded $I(q)$ versus q are shown, and all the details of the experiments and the results are reported in Table S2. The SLD of 73%-deuterated MexR (dMexR) matches the neutron SLD of 100% D_2O buffer (for further information, see Table S2). The SLD of DNA should match the 56% D_2O buffer (Table S2 and in Fig. S3 *E*). The dimensionless Kratky plots derived from these scattering curves are presented in Fig. S8, suggesting a well-folded MexR dimer and complex. To generate low-resolution structure envelopes of MexR-PII from SANS and SAXS data sets, we used MONSA (48,54). Resulting models consistently show a triangular-shaped MexR positioned around the DNA, in agreement with each monomer binding to the palindromic GTTGA sequence in two consecutive major grooves (Fig. 3 *B*, S7 and S6). The overlap of the DNA envelope over the protein envelope is probably due to the flexibility of the DNA and the similarity of the contrast in the SANS hydrogenated conditions.

Based on the obtained SAXS and SANS data sets, we were interested to see whether we could, at this resolution and ab initio, resolve any conformation differences between MexR in free and DNA-bound states. For this comparison, we decided to focus on the SAXS of apo-MexR and the SANS data of the d73MexR-PII at 56% v/v D_2O, which is the contrast MP of the DNA (Fig. S3 *E*); consequently, we predominantly detected the protein scattering contribution of the MexR bound state. Our Guinier analysis suggests R_gs of 23.2 Å and 23.7 Å (56 v/v % D_2O) for the unbound and bound states, respectively (Tables S1 and S2). In agreement, the *P(r)* distributions for the bound and unbound states indicate a similar D_{max} (74 and 65 Å respectively) but a slight redistribution of pairwise distances in possible agreement with slight compaction for the DNA-bound state compared with the free state (Fig. S8). However, the differences in R_g are small (22 and 23 Å) and could well be due to incomplete buffer matching and/or different effects of the hydration shell in SAS experiments and the effects of HD exchange within the solvent. Thus, it seems that by SAS alone, we are not able to detect a reliable, significant difference in dimensions for the MexR structures/ensembles in apo and DNA-bound states. Also, if there is a difference

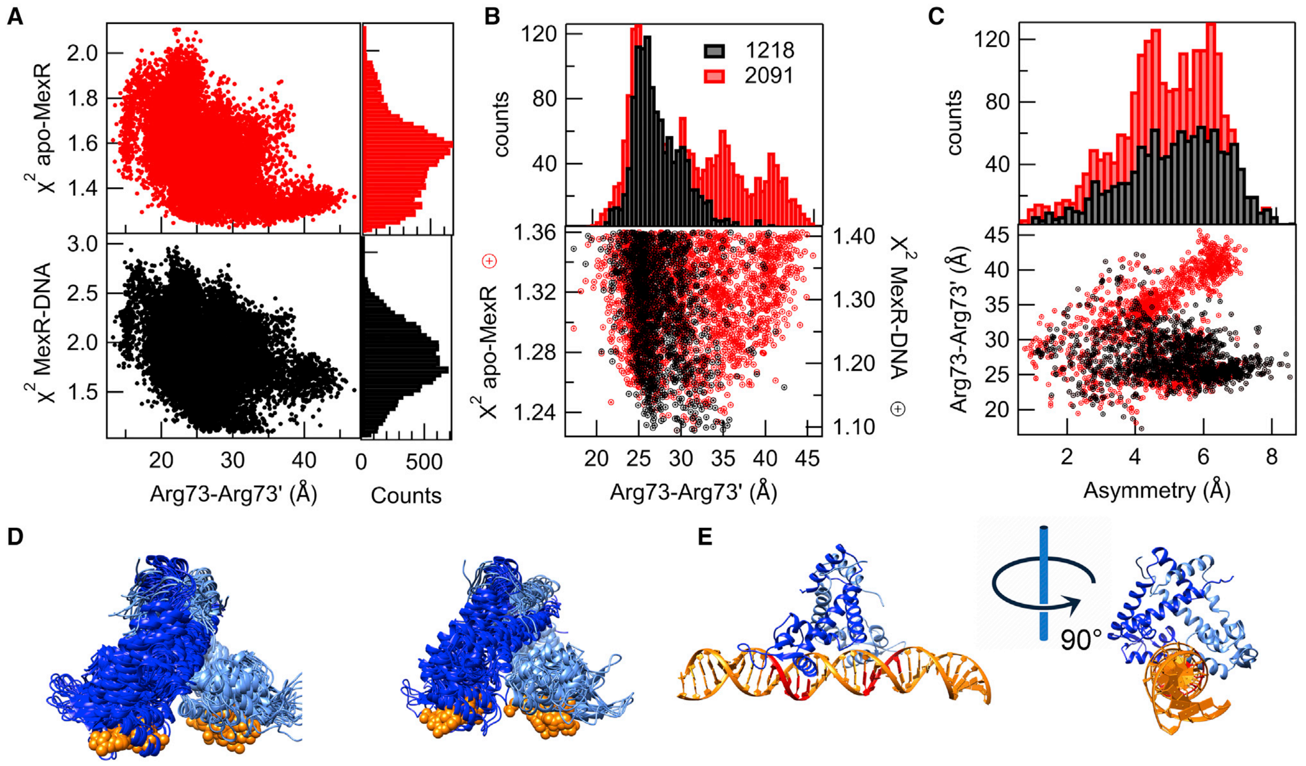

FIGURE 4 SAS-based molecular modeling. (*A*) Distribution of MD-derived MexR models as a function of the Arg73-Arg73′ distance and χ^2 to the SAXS data of the apo-protein (*red*) and the SANS data of dMexR-PII at 56% D_2O (*black*). Model counts show the propensities of models with best fit to data as a function of χ^2 to the corresponding data. (*B*) Magnification of best-fitting models (15% of total χ^2 range), colored as in (*A*), with model counts as a function of the Arg73-Arg73′ distances; the ensembles comprise 2091 structures for apo-MexR and 1218 structures for MexR when bound to DNA. 63% (770 structures) of the structures in the MexR-DNA ensemble are also members of the apo-MexR ensemble. (*C*) Scatter plot of the asymmetry of the best 15% χ^2 over the Arg73 distances. In red are the structures for the apo-MexR and in black the MexR in the presence of the DNA. From the scatter plot, it is evident that MexR in the presence of the DNA presents more asymmetry than in absence of the DNA. (*D*) Best-fitting ensembles representing apo-MexR and MexR when bound to DNA (MexR-DNA); 21/10 structures were chosen randomly from the 2091/1218 structures of the respective ensembles shown in (*B*)–(*C*). Monomeric units of the MexR are in blue/skyblue, and Arg73/Arg73′ sidechains are shown in orange spheres. (*E*) Best-fitting MexR-DNA complex to the combined SANS/SAXS data of MexR-DNA (overlaid with ab initio modeling in Fig. 4 *B*). The positioning of the palindromes is highlighted in red. To see this figure in color, go online.

in compactness between the protein in free and bound states, or ensembles of states, this is minimal or negligible.

SAS-based molecular modeling of MexR and the MexR-DNA complex

To increase the structural resolution of the SAS interpretation, we employed a forward modeling approach (55) using a structural ensemble comprising 15,000 MexR states, derived from MD simulations. We continued to use the SAXS of apo-MexR (Fig. 2) and the SANS data of the d73MexR-PII v/v 56% of D_2O to describe the free and DNA-bound MexR states, respectively. The closeness of fit to SAS data of structures within the MD ensemble as obtained by PEPSI-SAXS-SANS was analyzed as a function of the Arg73-Arg73′distance, which reflects the DNA-binding anchor residues and thus tentatively the position of the DNA-binding recognition helices (Fig. 4 *A*). The χ^2 minimum as a function of the Arg73-Arg73′distance is broad for both apo and DNA-bound MexR, but with lower number of well-fitting states for the DNA-bound MexR SAS data as judged by model counts (Fig. 4 *A*). To select the best-fitting ensemble of states for MexR-DNA and MexR-apo, a χ^2 cutoff corresponding to the lowest 15% of the total χ^2 range was applied, selecting 2091/1218 well-fitting conformations for the MexR-apo and MexR-DNA states, respectively (Fig. 4 *B*). The structural overlap between the two ensembles is significant: a majority (63%, 770 structures) of the SAS-derived DNA-bound MexR ensemble is also part of the apo-MexR SAS ensemble. Notably the level of dimer asymmetry (see materials and methods for definition) is pronouncedly higher in the ensemble of DNA-bound MexR states compared with the apo ensemble (Fig. 4 *C*). This higher degree of asymmetry as well as the structural spread of the well-fitting conformations is visualized in a representative ensemble of states in Fig. 4 *D*. We note that the ab initio DAMFILT beads model derived from the MexR-apo SAXS analysis appears to fit better with the

ensemble of apo solution states in Fig. 4 *D* than to the MexR-apo crystal structures (Fig. S11).

To obtain a SAS-based molecular model for the entire MexR-DNA complex, we selected three structures from the SAS-based ensemble of DNA-bound MexR, with a χ^2 of 1.20 with respect to SAS data, and respective R_gs and Arg73 distances of 21.77, 21.99, and 22.06 Å and 26.40, 30.04, and 31.28 Å. We first attempted to dock MexR to PII DNA in its B form using the software HDOCKlite v1.1 (45). However, we consistently got poor fits of the resulting models to both SAXS and SANS data, and by structural analysis, we found that this rigid-body docking was unable to dock the DNA-binding helices into the DNA, and too large complexes were thus systematically generated (Fig. S10). We then instead pursued a template-based docking, where we first aligned PII B-DNA to the DNA models in the OhrR-DNA (PDB:ID 1Z9C) and SlyA-DNA (PDB:ID 3QPT) crystal structures, using the dyad center as an MP. Each of the three selected DNA-bound MexR models were then aligned to OhrR and SlyA dimer, placing the MexR in an approximate DNA-bound location, and energy minimized allowing for flexibility both in the protein and in the DNA to resolve slight clashes. The complex model with best fit to all MexR-DNA SAS data was chosen to structurally represent the MexR-DNA complex (Fig. 4 *E*), and it is accommodated well within the ab initio SAS-reconstructed structure envelope (Fig. 3). Furthermore, this final model retains the observed asymmetry of the MexR-DNA bound ensemble (4C) as well as the overall linear shape of the PII DNA, with small adjustments in the protein-DNA contact areas as observed in both OhrR-DNA (14) and SlyA-DNA (19) crystal structures.

DISCUSSION

In this work, we have investigated the DNA-binding of MexR, a protein within the MarR family, with a particular focus on induced-fit binding compared with conformational selection (56). The prevailing induced-fit model for ligand binding in the MarR family implies two distinct structural states (apo and ligand bound), where ligand binding is essential to instigate the transition between the apo structure and that of the bound state. The ligand inducing the fit could be DNA itself, as proposed for MarR proteins OhrR and MepR (14,57), or metal ions, as in MarR family metalloregulatory proteins where metal binding stabilizes conformations readily amenable to DNA binding (reviewed in Reyes-Caballero et al. (58)).

MexR belongs to a group of MarR family proteins that do not need a ligand to bind DNA, but, once bound to DNA, they need to be ready to bind molecules that instigate repressor release. Jointly based on experimental data, where we trapped the crystal structure of a DNA-binding deficient MexR mutant, and extensive computational evaluation, we previously proposed a model for MexR ligand binding where variously populated structural subensembles within a wide apo ensemble of structures bind ligands such as DNA and/or regulators in a conformational selection mechanism (18). The advantage of such a response is that a single transcriptional regulator already in its apo state will showcase a wide range of structures, readily available for binding to DNA but also to multiple small molecules including clinically essential antibiotics that will release DNA binding (59). Thus, with a small genomic investment, a limited number of adaptable transcriptional regulators enables *P. aeruginosa* to recognize and initiate efflux for a wide range of toxic molecules in an efficient and versatile response. Conformational dynamics related to allosteric regulation have since been proposed for several MarR family members (60–62). However, direct experimental evidence supporting conformational selection as a mechanism for DNA binding in the absence of allosteric activation has not yet been shown.

To evaluate the structural envelope of the MexR-DNA complex as well as the envelopes of free and DNA-bound MexR ensembles in solution, we used neutron and x-ray SAS jointly with molecular modeling. Within experimental resolution, we could not resolve any closed-to-open DNA-binding transition based on R_g analysis alone, and the two states showed similar level of compactness. However, our SAS-based modeling showed that although a fairly wide range of MexR states were consistent with apo SAXS data, only a narrower ensemble of MexR states were consistent with the DNA-bound SANS data (Fig. 4 *B*). Importantly, a majority (63%) of the states in the DNA-bound MexR ensemble were also part of the larger MexR-apo ensemble. This suggests significant access to the DNA-bound states already in the absence of ligand, which for the first time to our knowledge provides experimental support for conformational selection as a major contributor to MexR-DNA binding in solution (62,63).

Interestingly, the SAS-selected ensemble of DNA-bound MexR is distinctly asymmetric, whereas the apo-MexR ensemble does not show such preference (Fig. 4 *C*). In our previous molecular dynamics (MD) simulations, we noted that there is an intrinsic asymmetric structural property within MexR, implied by the asymmetric packing of aromatic residues in the dimer interface, and which is interconvertible within the time range of the MD simulation (18). DNA binding may select for a distinct MexR asymmetry, which may also relate to our choice of a native binding site (PII), symmetric only within the palindromes (Fig. 1 *B*). DNA binding sites are normally symmetrized before co-crystallization with a protein target to obtain highest resolution. In reality, such symmetric DNA binding sites seldom occur due to overlap with other DNA binding sites such as promoter regions. The preference for asymmetry might be a distinct feature of MexR binding to native DNA sites in solution that may have been overlooked due to experimental limitations in the crystal. If, and how, DNA-binding dynamics (1) is related to this asymmetric preference, or whether the

fuzziness of the MexR-apo and MexR-DNA ensembles reflect intrinsic dynamic features of both bound and free complexes, remains to be investigated.

With the help of contrast matching, featured by neutrons, ab initio models revealed similar MexR positioning on DNA that has been observed for MarR family proteins OhrR and SlyA. In agreement, template-based docking using OhrR- and SlyA-DNA complexes provided MexR-PII-DNA models with excellent fit to experimental SAS data (Figs. 3 B and 4 *E*). Specifically, MexR appears to bind DNA in a conformation enabling wHTH domain binding to access the major grooves with the conserved palindromes similar to what has been observed for other MarR proteins, even if the distance between the palindromic sequences is longer in MexR (5 bp) than in OhrR (0 bp) or SlyA (2 bp). The final MexR-PII complex model retains the asymmetry present in the MexR-DNA ensemble, which may relate to the longer distances between the palindromes. The neutron scattering intensity for the DNA alone suggests a linear DNA, but the adaptability of B-DNA required to efficiently bind models from the MexR-DNA ensemble suggests a final induced-fit refinement of the conformational selection (56).

Taken together, our observations jointly support a conformational selection model for MexR-DNA binding with plausible asymmetric binding properties, although detailed structural features cannot be evaluated at this level of resolution. The SAXS/SANS approach can easily be extended to other protein-DNA complexes, where complex structures are not amenable to high-resolution structure determination. To our knowledge, this is the first time SANS has been used to study a MarR-type protein-DNA complex in solution, and this approach could well extend our understanding of the diversity of DNA binding within this protein family. Further unresolved questions remain in this field where SAS could contribute, such as the role of the two adjacent DNA-binding sites that are commonly found in operators regulated by MarR family proteins (64). An increased understanding of DNA binding and release mechanisms within the MarR family is essential to understand mechanisms for multiresistance and how these could be addressed therapeutically to improve worldwide health.

DATA AND CODE AVAILABILITY

The SAS data of apo MexR and MexR-PII complex with associated models are deposited in the Small-angle Scattering Biological Data Bank with the accession codes SASDMG9 and SASDMH9 (SASBDB: www.sasbdb.org) (65).

SUPPORTING MATERIAL

Supporting material can be found online at https://doi.org/10.1016/j.bpj.2022.11.2949.

AUTHOR CONTRIBUTIONS

F.C. and M.S. designed the research. F.C. and Z.P. performed research and analyzed data, V.M. and L.G.M. contributed biological and analytic tools, C.J., F.G., and A.M. supervised SAXS and SANS experiments and data analysis, and B.W. designed and supervised the molecular modeling. F.C., Z.P., B.W., and M.S. wrote the article with input from all authors. M.S. supervised the entire project. The authors declare no competing interests.

ACKNOWLEDGMENTS

We would like to acknowledge ILL for the allocated beam time at the D22 instrument (https://doi.org/10.5291/ILL-DATA.8-03-921) and EMBL Hamburg for an access to P12 bioSAXS beamline. Mass spectrometry analysis was carried out at the Mass Spectrometry Core Facility of Faculty of Medicine and Health Sciences, whereas ITC and SAXSess analysis were carried out at the ProLinC core facility of the Faculty of Technology, both at Linköping University, Sweden. PhD positions and experimental running costs were funded for F.C. by the ILL Graduate School (IGS), Grenoble, France and a project grant from the Swedish Research Council (VR 2018-04392, awarded to B.W., A.M. and M.S.), and for Z.P. by the Swedish Foundation for Strategic Research (SSF) within the Swedish national graduate school in neutron scattering SwedNess (GSn15-00 08). The synchrotron SAXS data were collected at beamline P12 operated by EMBL Hamburg at the PETRA III storage ring (DESY, Hamburg, Germany). We would like to thank Cy Jeffries for his assistance in using the beamline. This work was partially supported by the IDEX-IRS project PEPSI-SAS "Small-angle scattering using polynomial expansions" funded by University Grenoble Alpes (UGA), France. We would like to thank Sergei Grudinin, UGA, for helpful advice and discussion.

DECLARATION OF INTERESTS

The authors declare that they have no conflict of interest.

REFERENCES

1. Fuxreiter, M., I. Simon, and S. Bondos. 2011. Dynamic protein- DNA recognition: beyond what can be seen. *Trends Biochem. Sci.* 36:415–423. https://doi.org/10.1016/j.tibs.2011.04.006.
2. Wolberger, C. 2021. Howstructural biology transformed studies of transcription regulation. *J. Biol. Chem.* 296:100741. https://doi.org/10.1016/j.jbc.2021.100741.
3. Aravind, L., V. Anantharaman, ..., L. Iyer. 2005. The many faces of the helix-turnhelix domain: transcription regulation and beyond. *FEMS Microbiol. Rev.* 29:231–262. https://doi.org/10.1016/j.fmrre.2004.12.008.
4. Gajiwala, K. S., and S. K. Burley. 2000. Winged helix proteins. *Curr. Opin. Struct. Biol.* 10:110–116. https://doi.org/10.1016/S0959-440X(99)00057-3.
5. Mensa, J., J. Barberán, ..., A. Oliver. 2018. Antibiotic selection in the treatment of acute invasive infections by Pseudomonas aeruginosa: guidelines by the Spanish Society of Chemotherapy. *Rev. Esp. Quimioter.* 31:78–100.
6. Sommer, L. M., H. K. Johansen, and S. Molin. 2020. Antibiotic resistance in Pseudomonas aeruginosa and adaptation to complex dynamic environments. *Microb. Genom.* 6:mgen000370. https://doi.org/10.1099/mgen.0.000370.
7. Saito, K., S. Eda, ..., T. Nakae. 2001. Molecular mechanism of MexR-mediated regulation of MexAB-OprM efflux pump expression in Pseudomonas aeruginosa. *FEMS Microbiol. Lett.* 195:23–28. https://doi.org/10.1111/j.1574-6968. 2001.tb10492.x.

8. Grove, A. 2013. MarR family transcription factors. *Curr. Biol.* 23:R142–R143.

9. Deochand, D. K., and A. Grove. 2017. MarR family transcription factors: dynamic variations on a common scaffold. *Crit. Rev. Biochem. Mol. Biol.* 52:595–613. https://doi.org/10.1080/10409238.2017.1344612.

10. Beggs, G. A., R. G. Brennan, and M. Arshad. 2020. MarR family proteins are important regulators of clinically relevant antibiotic resistance. *Protein Sci.* 29:647–653. https://doi.org/10.1002/pro.3769.

11. Evans, K., L. Adewoye, and K. Poole. 2001. MexR repressor of the MexAB-oprM multidrug efflux operon of Pseudomonas aeruginosa: identification of MexR binding sites in the MexA-MexR intergenic region. *J. Bacteriol.* 183:807–812. https://doi.org/10.1128/JB.183. 3.807-812.2001.

12. Andrésen, C., S. Jalal, ..., M. Sunnerhagen. 2010. Critical biophysical properties in the Pseudomonas aeruginosa efflux gene regulator MexR are targeted by mutations conferring multidrug resistance. *Protein Sci.* 19:680–692. https://doi.org/10.1002/pro.343.

13. Lim, D., K. Poole, and N. C. J. Strynadka. 2002. Crystal structure of the MexR repressor of the MexRAB-oprM multidrug efflux operon of Pseudomonas aeruginosa. *J. Biol. Chem.* 277:29253–29259. https://doi.org/10.1074/jbc.M111381200.

14. Hong, M., M. Fuangthong, ..., R. G. Brennan. 2005. Structure of an OhrR-ohrA operator complex reveals the DNA binding mechanism of the MarR family. *Mol. Cell.* 20:131–141. https://doi.org/10.1016/j.molcel.2005.09.013.

15. Wilke, M. S., M. Heller, ..., N. C. J. Strynadka. 2008. The crystal structure of MexR from Pseudomonas aeruginosa in complex with its antirepressor ArmR. *Proc. Natl. Acad. Sci. USA.* 105:14832–14837. https://doi.org/10.1073/pnas.0805489105.

16. Chen, H., J. Hu, ..., C. He. 2008. The Pseudomonas aeruginosa multidrug efflux regulator MexR uses an oxidation-sensing mechanism. *Proc. Natl. Acad. Sci. USA.* 105:13586–13591. https://doi.org/10.1073/pnas.0803391105.

17. Chen, H., C. Yi, ..., C. He. 2010. Structural insight into the oxidation-sensing mechanism of the antibiotic resistance of regulator MexR. *EMBO Rep.* 11:685–690. https://doi.org/10.1038/embor.2010.96.

18. Anandapadamanaban, M., R. Pilstål, ..., M. Sunnerhagen. 2016. Mutation-induced population shift in the MexR conformational ensemble disengages DNA binding: a novel mechanism for MarR family derepression. *Structure.* 24:1311–1321. https://doi.org/10.1016/j.str.2016.06.008.

19. Dolan, K. T., E. M. Duguid, and C. He. 2011. Crystal structures of SlyA protein, a master virulence regulator of Salmonella, in free and DNA-bound states. *J. Biol. Chem.* 286:22178–22185. https://doi.org/10.1074/jbc.M111.245258.

20. Savitsky, P., J. Bray, ..., O. Gileadi. 2010. High-throughput production of human proteins for crystallization: the SGC experience. *J. Struct. Biol.* 172:3–13. https://doi.org/10.1016/j. jsb.2010.06.008.

21. Moore, P. B. 1979. The preparation of deuterated ribosomal materials for neutron scattering. *Methods Enzymol.* 59:639–655. https://doi.org/10.1016/0076-6879(79)59119-8.

22. Meilleur, F., K. L. Weiss, and D. A. A. Myles. 2009. Deuterium labeling for neutron structure-function-dynamics analysis. *In* Micro and Nano Technologies in Bioanalysis Springer, pp. 281–292. https://doi.org/10.1007/978-1- 59745-483-4_18.

23. Bradford, M. M. 1976. A rapid and sensitive method for the quantitation of microgram quantities of protein utilizing the principle of protein-dye binding. *Anal. Biochem.* 72:248–254. https://doi.org/10.1016/0003 - 2697(76)90527-3.

24. Blanchet, C. E., A. Spilotros, ..., D. I. Svergun. 2015. Versatile sample environments and automation for biological solution X-ray scattering experiments at the P12 beamline (PETRA III, DESY). *J. Appl. Crystallogr.* 48:431–443. https://doi.org/10.1107/S160057671500254X.

25. Panjkovich, A., and D. I. Svergun. 2018. CHROMIXS: automatic and interactive analysis of chromatography-coupled smallangle X-ray scattering data. *Bioinformatics.* 34:1944–1946. https://doi.org/10.1093/bioinformatics/btx846.

26. Trewhella, J., A. P. Duff, ..., A. E. Whitten. 2017. Publication guidelines for structural modelling of small-angle scattering data from biomolecules in solution: an update. *Acta Crystallogr. D Struct. Biol.* 73:710–728. https://doi.org/10.1107/S2059798317011597.

27. Grudinin, S., M. Garkavenko, and A. Kazennov. 2017. Pepsi- SAXS: an adaptive method for rapid and accurate computation of small-angle X-ray scattering profiles. *Acta Crystallogr. D Struct. Biol.* 73:449–464. https://doi.org/10.1107/S2059798317005745.

28. Mutti, P., F. Cecillon, ..., J. Ratel. 2011. Nomad more than a simple sequencer. *In* Proc. ICALEPCS..

29. Dewhurst, C. 2007. GRASP: Graphical Reduction and Analysis SANS Program for Matlab". Institut Laue-Langevin (2001–2007).

30. Grillo, I. 2008. Small-angle neutron scattering and Applications in soft condensed matter. *In* Soft Matter Characterization. R. Borsali and R. Pecora, eds Dordrecht: Springer Netherlands, pp. 723–782. https://doi.org/10.1007/978-1-4020- 4465-6_13.

31. Larsen, A. H., and M. C. Pedersen. 2021. Experimental noise in small-angle scattering can be assessed using the Bayesian indirect Fourier transformation. *J. Appl. Crystallogr.* 54:1281–1289. https://doi.org/10.1107/S1600576721006877.

32. Pesce, F., and K. Lindorff-Larsen. 2021. Refining conformational ensembles of flexible proteins against small-angle x-ray scattering data. *Biophys. J.* 120:5124–5135. https://doi.org/10.1016/j.bpj.2021.10.003.

33. Guinier, A., G. Fournet, and K. L. Yudowitch. 1955. Small-angle Scattering of X-Rays. Wiley New York.

34. Kline, S. R. 2006. Reduction and analysis of SANS and USANS data using IGOR Pro. *J. Appl. Crystallogr.* 39:895–900. https://doi.org/10.1107/S0021889806035059.

35. Vad, T., and W. F. C. Sager. 2011. Comparison of iterative desmearing procedures for one-dimensional small-angle scattering data. *J. Appl. Crystallogr.* 44:32–42. https://doi.org/10.1107/S0021889810049721.

36. Anandapadamanaban, M., N. C. Kyriakidis, ..., M. Sunnerhagen. 2019. E3 ubiquitin-protein ligase TRIM21-mediated lysine capture by UBE2E1 reveals substrate-targeting mode of a ubiquitin-conjugating E2. *J. Biol. Chem.* 294:11404–11419. https://doi.org/10.1074/jbc.RA119.008485.

37. Petoukhov, M. V., D. Franke, ..., D. I. Svergun. 2012. New developments in the ATSAS program package for small-angle scattering data analysis. *J. Appl. Crystallogr.* 45:342–350. https://doi.org/10.1107/S0021889812007662.

38. Svergun, D. I. 1992. Determination of the regularization parameter in indirect-transform methods using perceptual criteria. *J. Appl. Crystallogr.* 25:495–503. https://doi.org/10.1107/S0021889892001663.

39. Whitten, A. E., S. Cai, and J. Trewhella. 2008. MULCh: modules for the analysis of small-angle neutron contrast variation data from biomolecular assemblies. *J. Appl. Crystallogr.* 41:222–226. https://doi.org/10.1107/S0021889807055136.

40. IbeL, K., and H. B. Stuhrmann. 1975. Comparison of neutron and X-ray scattering of dilute myoglobin solutions. *J. Mol. Biol.* 93:255–265. https://doi.org/10.1074/jbc.M405586200.

41. Franke, D., and D. I. Svergun. 2009. DAMMIF, a program for rapid ab-initio shape determination in small-angle scattering. *J. Appl. Crystallogr.* 42:342–346. https://doi.org/10.1107/S0021889809000338.

42. Volkov, V. V., and D. I. Svergun. 2003. Uniqueness of ab initio shape determination in small-angle scattering. *J. Appl. Crystallogr.* 36:860–864. https://doi.org/10.1107/S0021889803000268.

43. Konarev, P. V., M. V. Petoukhov, and D. I. Svergun. 2016. Rapid automated superposition of shapes and macromolecular models using spherical harmonics. *J. Appl. Crystallogr.* 49:953–960. https://doi.org/10.1107/S1600576716005793.

44. Pronk, S., S. Páll, ..., E. Lindahl. 2013. Gromacs 4.5: a high-throughput and highly parallel open source molecular simulation toolkit. *Bioinformatics.* 29:845–854. https://doi.org/10.1093/bioinformatics/btt055.

45. Huang, S. Y., and X. Zou. 2014. A knowledge-based scoring function for protein-RNA interactions derived from a statistical mechanics-based

iterative method. *Nucleic Acids Res.* 42:e55. https://doi.org/10.1093/nar/gku077.

46. Tyka, M. D., D. A. Keedy, ..., D. Baker. 2011. Alternate states of proteins revealed by detailed energy landscape mapping. *J. Mol. Biol.* 405:607–618. https://doi.org/10.1038/nchembio. 232.

47. Clifton, L. A., C. L. Johnson, ..., J. H. Lakey. 2012. Lowresolution structure and dynamics of a colicin-receptor complex determined by neutron scattering. *J. Biol. Chem.* 287:337–346. https://doi.org/10.1074/jbc.M111.302901.

48. Svergun, D. I. 1999. Restoring lowresolution structure of biological macromolecules from solution scattering using simulated annealing. *Biophys. J.* 76:2879–2886. https://doi.org/10.1016/S0006-3495(99)77443-6.

49. André, I., C. E. M. Strauss, ..., D. Baker. 2008. Emergence of symmetry in homooligomeric biological assemblies". English. *Proc. Natl. Acad. Sci. USA.* 105:16148. https://doi.org/10.1073/pnas.0807576105.

50. Schindler, B. D., S. M. Seo, ..., G. W. Kaatz. 2015. Mutations within the MepA operator affect binding of the MepR regulatory protein and its induction by MepA substrates in Staphylococcus aureus. *J. Bacteriol.* 197:1104–1114. https://doi.org/10.1128/JB.02558-14.

51. Wilkinson, S. P., and A. Grove. 2006. Ligand-responsive transcriptional regulation by members of the MarR family of winged helix proteins. *Curr. Issues Mol. Biol.* 8:51–62. https://doi.org/10.21775/cimb.008.051.

52. Martin, R. G., and J. L. Rosner. 1995. Binding of purified multiple antibiotic-resistance repressor protein (MarR) to mar operator sequences. *Proc. Natl. Acad. Sci. USA.* 92:5456–5460. https://doi.org/10.1073/pnas.92. 12.5456.

53. Wilkinson, S. P., and A. Grove. 2004. HucR, a novel uric acidresponsive member of the MarR family of transcriptional regulators from Deinococcus radiodurans. *J. Biol. Chem.* 279:51442–51450. https://doi.org/10.1074/jbc.M405586200.

54. Franke, D., M. V. Petoukhov, ..., D. I. Svergun. 2017. Atsas 2.8: a comprehensive data analysis suite for small-angle scattering from macromolecular solutions. *J. Appl. Crystallogr.* 50:1212–1225. https://doi.org/10.1107/S1600576717007786.

55. Thomasen, F. E., and K. Lindorff-Larsen. 2021. Conformational ensembles of intrinsically disordered proteins and flexible multidomain proteins. preprint at arXiv. https://doi.org/10.1042/BST20210499.

56. Boehr, D. D., R. Nussinov, and P. E. Wright. 2009. The role of dynamic conformational ensembles in biomolecular recognition. *Nat. Chem. Biol.* 5:789–796. https://doi.org/10.1038/nchembio.232.

57. Birukou, I., S. M. Seo, ..., R. G. Brennan. 2014. Structural mechanism of transcription regulation of the Staphylococcus aureus multidrug efflux operon mepRA by the MarR family repressor MepR. *Nucleic Acids Res.* 42:2774–2788. https://doi.org/10.1093/nar/gkt1215.

58. Reyes-Caballero, H., G. C. Campanello, and D. P. Giedroc. 2011. Metalloregulatory proteins: metal selectivity and allosteric switching. *Biophys. Chem.* 156:103–114. https://doi.org/10.1016/j.bpc.2011.03.010.

59. Tung, Q. N., T. Busche, ..., H. Antelmann. 2020. The redox-sensing MarR-type repressor HypS controls hypochlorite and antimicrobial resistance in Mycobacterium smegmatis. *Free Radic. Biol. Med.* 147:252–261. https://doi.org/10.1016/j. freeradbiomed.2019.12.032.

60. Liguori, A., E. Malito, ..., M. J. Bottomley. 2016. Molecular basis of ligand-dependent regulation of NadR, the transcriptional repressor of meningococcal virulence factor NadA. *PLoS Pathog.* 12:e1005557. https://doi.org/10.1371/journal.ppat 1005557.

61. Kim, Y., G. Joachimiak, ..., A. Joachimiak. 2016. How aromatic compounds block DNA binding of HcaR catabolite regulator. *J. Biol. Chem.* 291:13243–13256. https://doi.org/10.1074/jbc. M115.712067.

62. Capdevila, D. A., F. Huerta, ..., D. P. Giedroc. 2018. Tuning site-specific dynamics to drive allosteric activation in a pneumococcal zinc uptake regulator. *Elife.* 7:e37268. https://doi.org/10.7554/eLife.37268.

63. Arunkumar, A. I., G. C. Campanello, and D. P. Giedroc. 2009. Solution structure of a paradigm ArsR family zinc sensor in the DNA-bound state. *Proc. Natl. Acad. Sci. USA.* 106:18177–18182. https://doi.org/10.1073/pnas. 0905558106.

64. Will, W. R., and F. C. Fang. 2020. The evolution of MarR family transcription factors as counter-silencers in regulatory networks. *Curr. Opin. Microbiol.* 55:1–8. https://doi.org/10.1016/j.mib.2020.01.002.

65. Kikhney, A. G., C. R. Borges, ..., D. I. Svergun. 2020. SASBDB: towards an automatically curated and validated repository for biological scattering data. *Protein Sci.* 29:66–75. https://doi.org/10.1002/pro.3731.

Biophysical Journal, Volume *122*

Supplemental information

Small-angle x-ray and neutron scattering of MexR and its complex with DNA supports a conformational selection binding model

Francesca Caporaletti, Zuzanna Pietras, Vivian Morad, Lars-Göran Mårtensson, Frank Gabel, Björn Wallner, Anne Martel, and Maria Sunnerhagen

SUPPLEMENTARY MATERIAL OF SMALL-ANGLE X-RAY AND NEUTRON SCATTERING OF MEXR AND ITS COMPLEX WITH DNA SUPPORTS A CONFORMATIONAL SELECTION BINDING MODEL

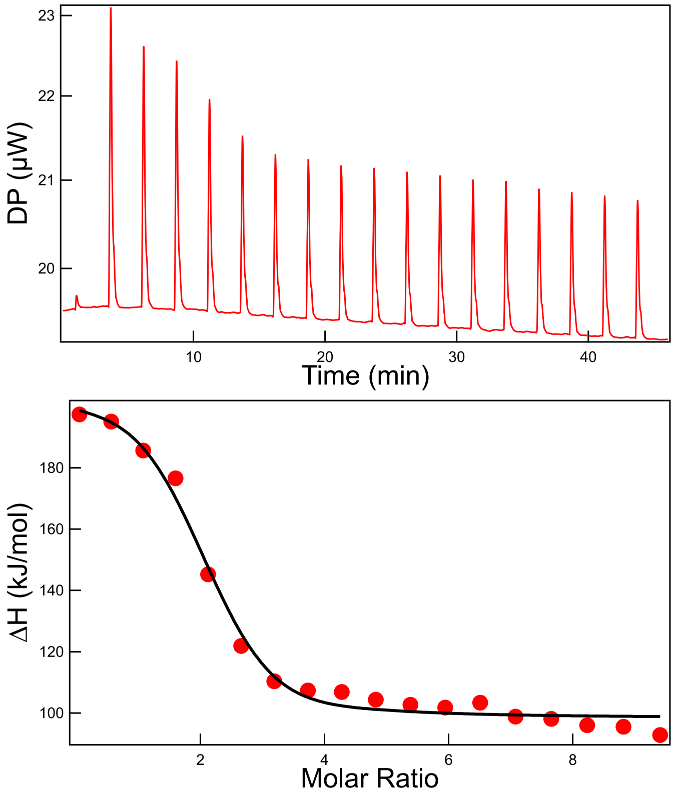

K_D ($10^{-9}M$)	N_s	ΔH (kJ/mol)	ΔG (kJ/mol)	$-T\Delta S$ (kJ/mol)
240 ±60	1.91 ±0.06	105 ±5	-37.2	-142

Figure S1: **ITC profiles for the binding of MexR to PII DNA.** The upper panel presents the thermogram for MexR titration into double stranded PII DNA solution. The bottom panel shows the heat evolution of the added MexR to PII DNA. Data (red circles) were fitted to the "one set of sites" model, and the solid black line represents the best fit. The table reports the thermodynamic parameters for the association of MexR with the PII DNA segment. Each column represents the result of the fit: K_D: dissociation constant, N_s: number of sites, ΔH: enthalpy, ΔG: free enthalphy of Gibbs, -TΔS: entropy

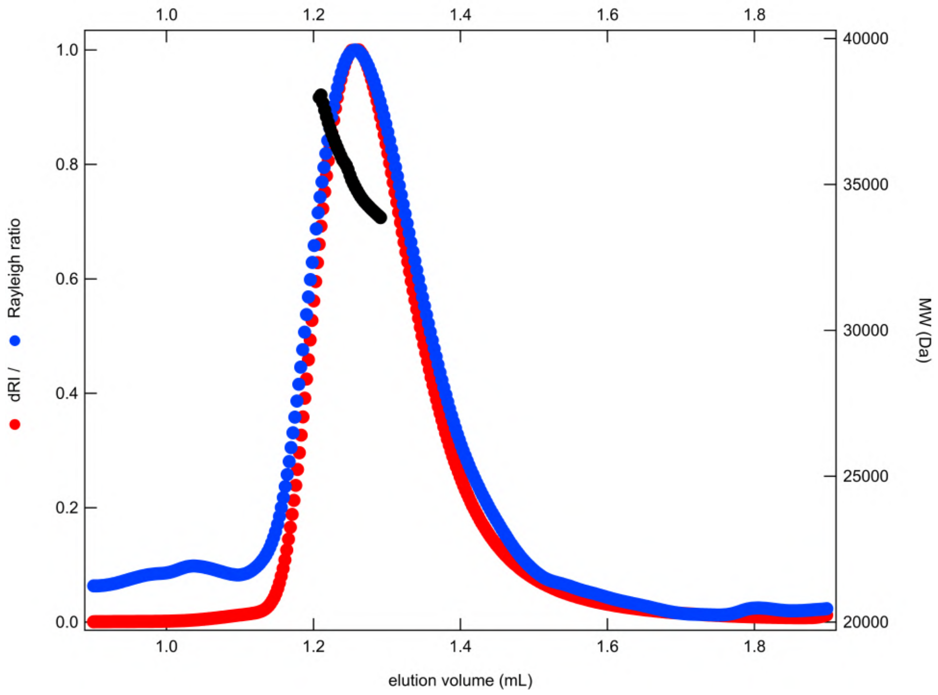

Figure S2: **The MALLS (blue) and dRI (red) traces acquired for the SEC of MexR protein.** The MW distribution through the MexR peak is shown as a black line

Table S1: Resume table of results and conduction for apo-MexR. The tables are inspired by the guidelines described in Trewhella *et at.* (26).

**SAMPLE**	apo-MexR
**ORGANISM**	*P. aeruginosa*
**SOURCE**	produced at LiU
**UNIPROT ID**	P52003
**AMINO ACID SEQUENCE**	SM VNPDLMPALMAVFQHVRTRIQ SELDCQRLDLTPPDVHVLKLIDEQRGLNLQDLGRQMCRDKAL ITRKIRELEGRNLVRRERNP SDQRSFQLFLTDEGLAIHQHAEAIMSRVHDELFAPLTPVEQATL VHLLDQCL
**EXTINCTION COEFFICIENT AT 280 nm** ($M^{-1}cm^{-1}$)	8.00 10^{-3}
**PARTIAL SPECIFIC VOLUME** (cm^3g^{-1})	0.74
**STOICHIOMETRY**	2
**MONOMER VOLUME FROM CHEMICAL COMPOSITION** (Å^3)	18600
**MONOMER MASS FROM CHEMICAL COMPOSITION (KDa)**	15.3
**SOLVENT COMPOSITION**	20 mM HEPES, pH 7.1, 150 mM NaCl, 10 mM DTT, 1% v/v glycerol
**SLD SAMPLE** (10^{-6}**Å**$^{-2}$)	12.3
**SLD SOLVENT** (10^{-6}**Å**$^{-2}$)	9.4
**CONTRAST** (10^{-6}**Å**$^{-2}$)	2.9

Table S1 continued from previous page

SAMPLE	apo-MexR
**SEC COLUMN**	S75 Increase 5/150
**LOADING CONCENTRATION** ($mg\,ml^{-1}$)	8.5
**INJECTION VOLUME**	35 μl
**FLOW RATE** ($ml\,min^{-1}$)	0.35
**SAXS data collection parameters**	
**SOURCE**	Petra III U29 undulator
**WAVELENGTH**	1.24 Å
**BEAM GEOMETRY AT SAMPLE**	0.99 mm capillary
**BEAM SIZE AT THE DETECTOR**	0.2 × 0.05 mm^2
**DETECTOR**	Pilatus 6M
**SAMPLE TO DETECTOR DISTANCE (m)**	3 (position 2)
**q-RANGE (Å^{-1})**	0.0024–0.73
**EXPOSURE TIME/NUMBER OF FRAMES**	1s/2880
**SAMPLE TEMPERATURE**	20 °C
**MONITORING FOR RADIATION DAMAGE**	SEC-SAXS
**MALLS**	Wyatt Technologies Mini-Dawn TREOS with an in-built quasi elastic light scattering (QELS) module coupled to an OptiLab T-Rex refractometer
**RI**	0.185 $ml\,g^{-1}$
**Software**	
**SAMPLE INJECTION**	ChemStation/BECQUEREL
**SAS DATA REDUCTION**	The SASFLOW pipeline incorporating RADAVER from the ATSAS 3.0 suite and CHROMIXS from the ATSAS 3.0
**CALCULATION OF ϵ FROM SEQUENCE**	EXPASY
**CALCULATION OF SLD FROM CHEMICAL COMPOSITION**	MULCh from The University of Sydney (39)
**STRUCTURAL PARAMETER**	$P(r)$ inversion with ATASAS/GNOM<br>Guinier analysis with PRIMUS;<br>Porod volume: GNOM
**BEAD MODELING**	DAMMIF
**ATOMIC STRUCTURE MODELING**	Molecular dynamics with Gromacs 4.5.5
**MODELING OF MISSING SEQUENCE FROM PDB FILE**	Modeller 9.13
**MOLECULAR GRAPHICS**	CHIMERA USFC
**Structural parameter**	
**GUINIER ANALYSIS (RECIPROCAL SPACE)**	
**I(0)** (cm^{-1})	0.0066 ± 0.0002
R_g **(Å)**	23.21 ± 0.12
sR_g **RANGE**	0.25 - 1.30
**FIDELITY**	0.2
**MW FROM ATSAS (Qp) (Da)**	26900
**MW FROM ATSAS (MoW) (Da)**	24700
**MW FROM ATSAS (Vc) (Da)**	32100
**MW FROM ATSAS (Size and Shape) (Da)**	34700
**MW FROM ATSAS (Bayesian Inference) (Da)**	31700
**MW FROM MALLS (kDa)**	34-38
**MW EXPECTED VALUE, DIMER (kDa)**	30.6
***P(r)* ANALYSIS (REAL SPACE)**	
**I(0)** (cm^{-1})	0.0067 ± 0.0002
**APPARENT R_g (Å)**	23.4
D_{max} **(Å)**	77 ± 5

Table S1 continued from previous page

SAMPLE	apo-MexR
**q-RANGE ($Å^{-1}$)**	0.010 - 0.34
**GNOM QUALITY**	0.95
**POROD VOLUME (Å)**	55700

Table S2: Resume table of results and conduction for MexR-PII. The tables are inspired by the guidelines described in J. et al. [26].

**SAMPLE**	MexR-PII					
	dMexR-PII 0%D_2O	dMexR-PII 56%D_2O	dMexR-PII 89%D_2O	hMexR-PII 0%D_2O	hMexR-PII 79%D_2O	MexR-PII
**ORGANISM**	*Pseudomonas aeruginosa*					
**SOURCE**	Protein: produced at LiU: both hydrogenated and 73% deuterated with *E. Coli* DNA:EUROGENETEC: SON: 1000831043					
**UNIPROT ID SEQUENCE**	Protein: P52003					
**NUCLEIC COMPONENT**	FW: 5’ - CTT ATT TTA GTT GAC CTT ATC AAC CTT GTT TCA G - 3’ RV: 5’ - CTG AAA CAA GGT TGA TAA GGT CAA CTA AAA TAA G - 3’					
**AMINO ACID SEQUENCE**	SM VNPDLMPALMAVFQHVRTRIQSELD CQRLDLTPPDVHVLKLIDEQRGLNLQDLGRQMCRDKAL ITRKIRELEGRNLVRRERNP SDQRSFQLFLTDEGLAIHQHAEAIMSRVHDELFAPLTPVEQATL VHLLDQCL					
**EXTINCTION COEFFICIENT** ($mL\, mg^{-1} cm^{-1}$)	Protein: 8.0010^{-3}(280 nm) DNA: 25.4 (260nm)					
**PARTIAL SPECIFIC VOLUME** ($cm^3 g^{-1}$)	0.681					
**STOICHIOMETRY**	2 MexR: 1RV: 1FW					
**COMPLEX VOLUME FROM CHEM. COMPOSITION ($Å^3$)**	60200					
**COMPLEX MASS FROM CHEM. COMPOSITION (Da)**	52120.84					
**CONCENTRATION ($mg\, ml^{-1}$)**	6 ± 1					3.1 ± 0.7
**SOLVENT COMPOSITION**	20 mM sodium phosphate buffer pH=7.1; 150 mM NaCl; 10 mM DTT					
**SLD SAMPLE** (10^{-6} $Å^{-2}$)	4.50	5.11	5.47	2.29	3.14	13.1
**SLD SOLVENT** (10^{-6} $Å^{-2}$)	-0.560	3.33	5.62	-0.560	4.93	9.40
**CONTRAST** ($10^{-6}Å^{-2}$)	5.06	1.78	-0.157	2.85	-1.79	3.74
**CONTRAST PROTEIN** (10^{-6} $Å^{-2}$)	5.74	2.59	0.74	2.36	-2.08	2.85
**CONTRAST DNA** (10^{-6} $Å^{-2}$)	3.78	0.23	-1.89	3.79	-1.86	5.45
**SAS data collection parameters**						
**EXPERIMENTAL SOURCE**	D22 at ILL					SAXess (Anton Paar)
**WAVELENGTH (Å)**	6 ± 0.6					0.15418
**BEAM GEOMETRY AT SAMPLE (mm)**	7 x10					10, slit
**SOURCE GEOMETRY (mm)**	40 x 55					10, slit

Table S2 continued from previous page

SAMPLE	MexR-PII					
	dMexR-PII 0%D_2O	dMexR-PII 56%D_2O	dMexR-PII 89%D_2O	hMexR-PII 0%D_2O	hMexR-PII 79%D_2O	MexR-PII
**COLLIMATOR L (m): SAMPLE DETECTOR D (m)**	1st: 5.6 : 5.6 2nd: 1.6 : 2.8					0.5 : 0.5
**q-RANGE (Å^{-1})**	0.014 - 0.5					0.014 - 0.5
**ABSOLUTE SCALING METHOD**	Direct: normalisation by flux at the sample position					Normalisation over empty beam peak
**BASIS FOR NORMALISATION TO CONSTANT COUNT**	Normalisation to monitor count					
**EXPOSURE TIME (min)**	41<br>15	31<br>15	71<br>30	41<br>30	42<br>15	4 x 20
**SAMPLE CONFIGURATION INCLUDING PATH LENGTH**	in 1 mm thickness Suprasil Quartz rectangular Hellma cuvettes					capillary diameter=1mm
**SAMPLE TEMPERATURE**	10 °C					
**Software**						
**SAS DATA REDUCTION**	GRASP and NCNR Macro for IGOR					SAXS quant 1D
**CALCULATION OF ϵ FROM SEQUENCE**	EXPASY for protein and OLIGOCALC for the DNAs					
**CALCULATION OF ρ FROM CHEMICAL COMPOSITION**	BSLDC from ISIS: http://psldc.isis.rl.ac.uk/Psldc<br>MULCH from the University of Sydney: http://smb-research.smb.usyd.edu.au/NCVWeb/ (39)					
**STRUCTURAL PARAMETERS**	*P(r)* inversion using ATSAS/GNOM;<br>Guinier analysis using ATSAS/Primus and NCNR macro;<br>Porod Volume using ATSAS/GNOM					
**BEAD MODELING**	ATSAS/MONSA					
**ATOMIC STRUCTURE MODELING**	Molecular Dynamics with Gromacs 4.5.5					
**MODELING OF MISSING SEQUENCE FROM PDB FILE**	Modeller 9.13					
**MOLECULAR GRAPHICS**	UCSF Chimera					
**Structural parameters**						
**GUINIER ANALYSIS (RECIPROCAL SPACE)[1]**						
**$I(0)_{theorical\ 1mg/ml}$ (cm^{-1})**	0.098	0.0061	0.0017	0.034	0.013	0.052
**$I(0)_{theorical}$ experimental concentration (cm^{-1})**	0.59	0.037	0.010	0.21	0.078	0.16
**I(0) (cm^{-1})**	0.66±0.01	0.038 ± 0.0008	0.010 ± 0.0005	0.21 ± 0.0009	0.081 ± 0.0007	0.191 ± 0.005
**R_g (Å)**	26.4 ± 0.5	23.7 ± 0.6	22 ± 2	28.9 ± 0.2	19.9 ± 0.4	27.7 ± 0.8
qR_g	0.50 - 1.3	0.44 - 1.3	0.46 - 1.2	0.51 - 1.3	0.45 - 1.3	0.48 - 1.3
χ^2	0.11	0.059	0.099	1.1	0.29	0.11
**MW FROM I(0) (Da)**	55600	28200	20800	54200	53400	52400
**MW EXPECTED FROM THE CHEM. COMPOSITION (Da)**	52200	31700		52200	52200	52200
***P(r)* ANALYSIS (REAL SPACE)**						

[1]This part is done with the NCNR IGOR Macro (34)

Table S2 continued from previous page

SAMPLE	MexR-PII					
	dMexR-PII 0%D_2O	dMexR-PII 56%D_2O	dMexR-PII 89%D_2O	hMexR-PII 0%D_2O	hMexR-PII 79%D_2O	MexR-PII
**I(0)** (cm^{-1})	0.680 ± 0.008	0.0383 ± 0.0006	0.0087 ± 0.0003	0.213 ± 0.001	0.081 ± 0.007	0.191 ± 0.002
**apparent R_g (Å)**	26.9 ± 0.6	24.1 ± 0.6	23 ± 2	29.5 ± 0.3	19.8 ± 0.4	27.9 ± 0.4
**D_{max} (Å)**	87-120	84-108	105 - 121	98 - 125	70 - 96	91-115
**q-RANGE (Å^{-1})**	0.016 - 0.30	0.016 - 0.33	0.046 - 0.50	0.015 - 0.28	0.015 - 0.42	0.018 - 0.27
**GNOM QUALITY**	0.79	0.76	0.52	0.72	0.80	0.69
**VOLUME THEORETIC**	60177.2					
**POROD VOLUME**	55500	43600	40600	76700	59300	51000
**Shape modeling results**						
**SYMMETRY ASSUMPTION**	P1					
**χ^2 MONSA**	5.3	5.6	3.8	9.7	8.0	1.5
**I(0) MONSA/I(0)$_{experimental}$**	0.93	0.93	0.33	1.0	0.94	1.0
**q-RANGE FOR FITTING (Å^{-1})**	0.018 - 0.25					

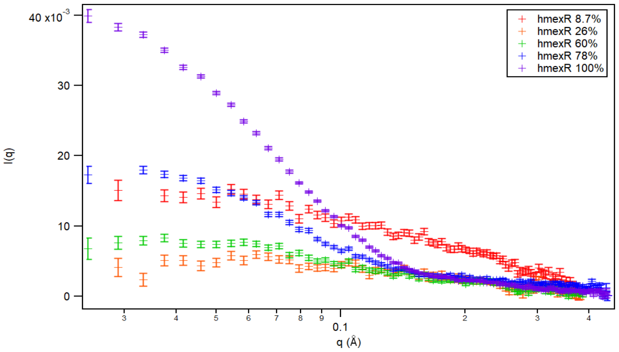

(a) Data set of hMexR.

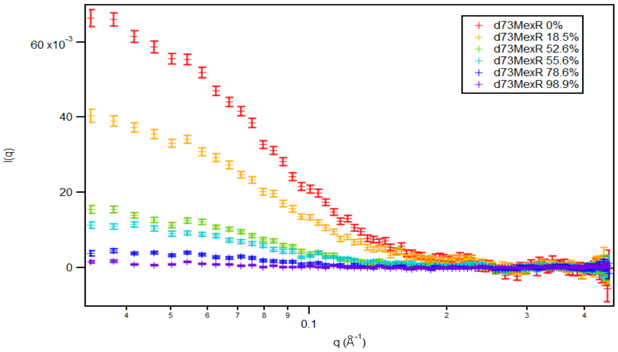

(b) Data set of d73MexR.

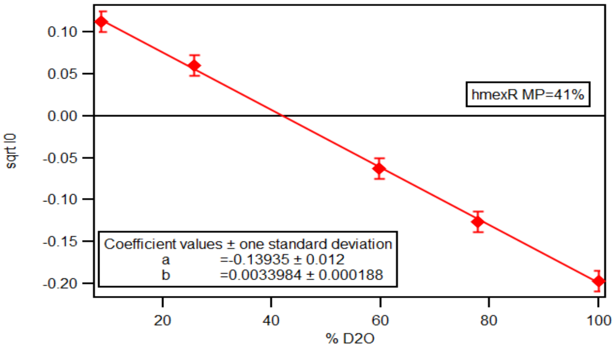

(c) Linear fit for the measurement of MP of hMexR.

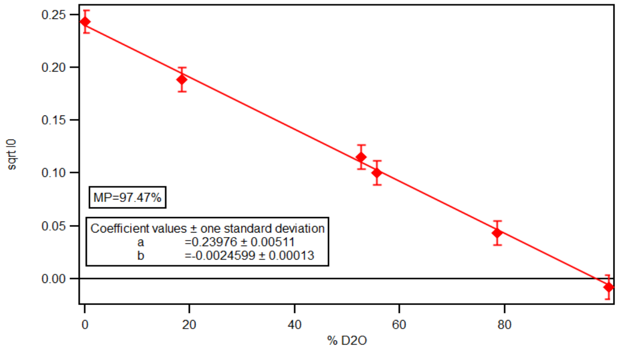

(d) Linear fit for the measurement of MP of d73MexR.

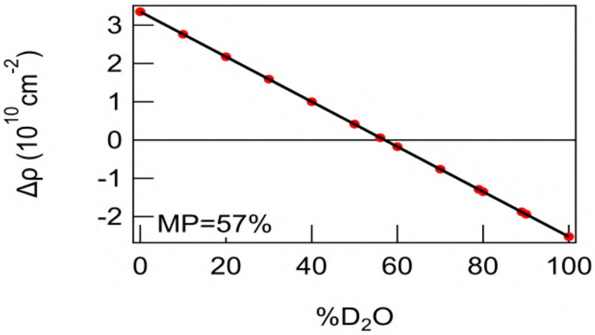

(e) Theorical MP for DNA

Figure S3: **Experimental determination of hMexR and d73MexR match points and theorical for PII DNA.** Panels a and b show the data sets of hMexR and d73mexR at different %D_2O. We acquired the data at D22 with a 2 m detector distance setup, and all samples analysed in this experiment were at 6 mg ml^{-1}. For the measure of the MPs, we performed a linear fit over $\sqrt{I_0}$ vs %D_2O. From the linear fit the MP are extract and they are respectively 41% and 97%. We reduced the data with GRASP software, and the analysis was performed using IGOR Pro v.7.

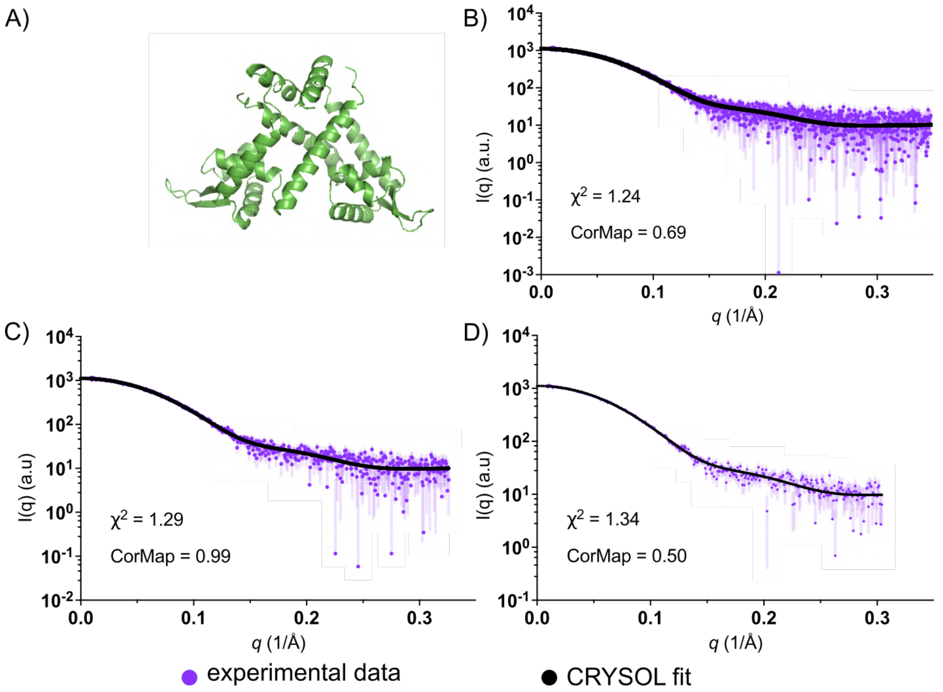

Figure S4: **Examples of re-binned data sets.** The errors are shown in light purple. A. sREFLEX refined model based on 1LNW PDB entry, amino acids Val5-Leu139. B. Scattering profile, presented as I(q) vs q collected at P12 BioSAXS beamline (EMBL-HH). The 2D-to-1D data reduction, including radial averaging, was performed using the SASFLOW pipeline incorporating RADAVER (ATSAS 2.8). 1249 data points. C. Re-binned data using DATREGRID, 624 data points D. Re-binned data using DATREGRID, 416 data points.

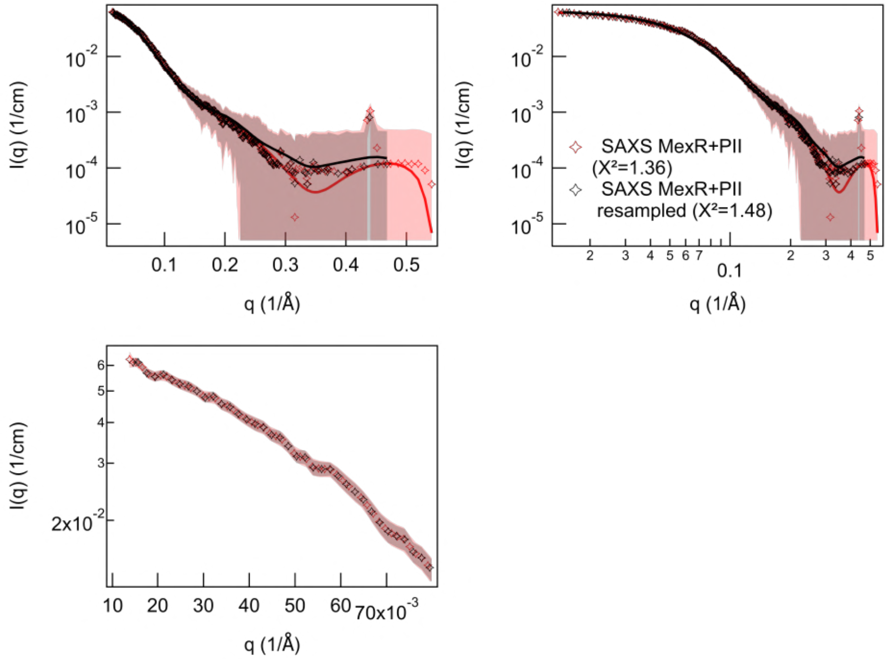

Figure S5: **Binning of SAXS data for MexR+PII.** The top panels show the data before (red) and after (black) the rebinning in both Log(I) vs log(q) and log(I) *vs.* q. The lines represent the results of the PEPSI-SAXS fits of the structure in Figure 3B on the original paper. The bottom panel shows a zoom at high q-values. In all figures, the error bars are represented as a semitransparent continuous band to help the visualisation of the point and the error bands.

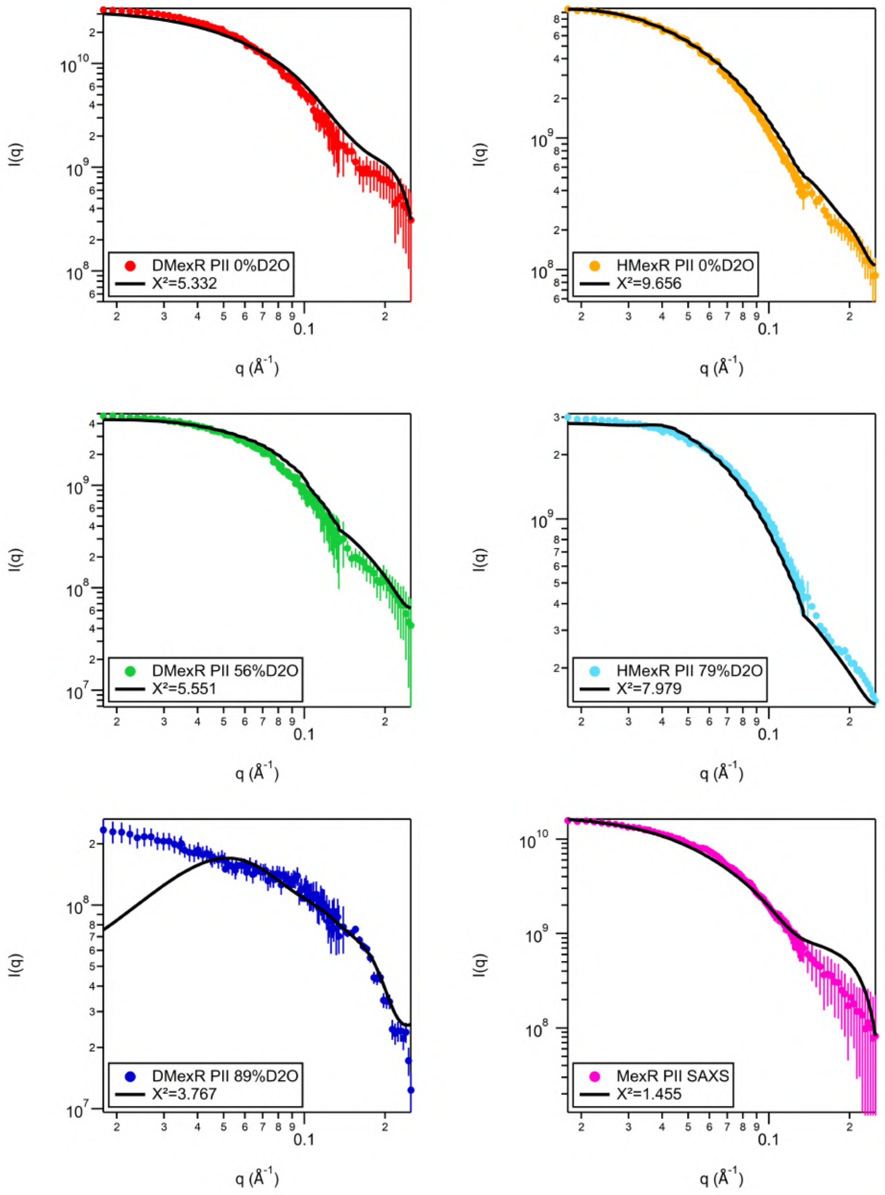

Figure S6: **SANS data set of MexR-PII complex supplemented with laboratory SAXS data of fully-protonated MexR-PII with MONSA fit.** The graphs show the reduced data of the experiment at D22 of the MexR bound with PII DNA. The black lines passing through the experimental point is the MONSA fit of the structure shown in figure 3B. Colorcode: SAXS of fully protonated complex (pink), SANS of fully protonated in 0% (yellow) and 79 % (cyan) D_2O buffers, and SANS of the dMexR-PII complex in 0 % (red), 56 % (green) and 89 % (navy) D_2O buffers. The shown data are the direct outcome from MONSA and they have a difference in intensity, because the output file of MONSA is the fit of the simulated scattering curve versus a smoothed-out version of the real-data multiply by a constant.

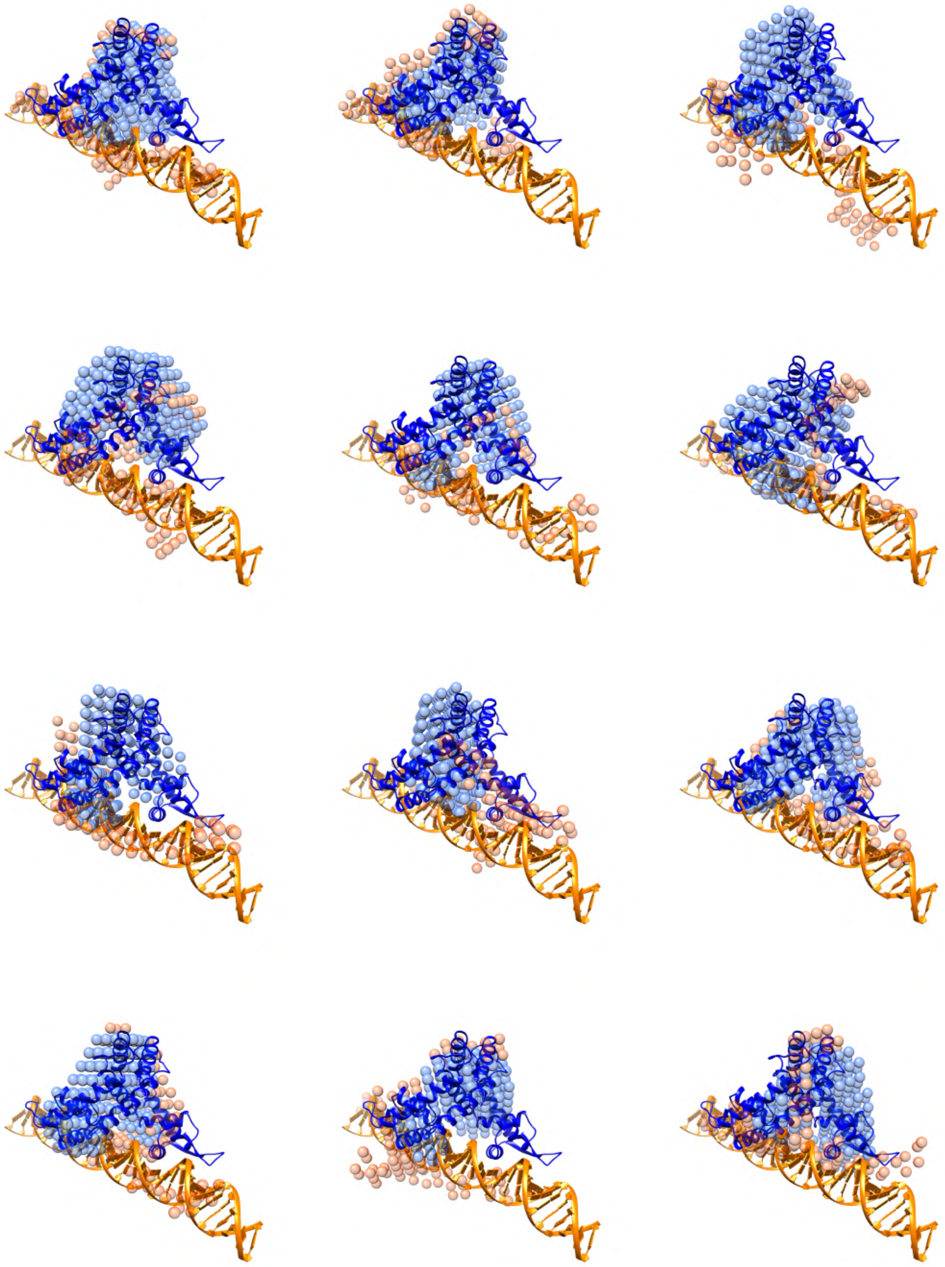

Figure S7: **MONSA for MexR-PII DNA.** Twelve results of MONSA *ab-initio* analysis performed to the MexR-PII data set.

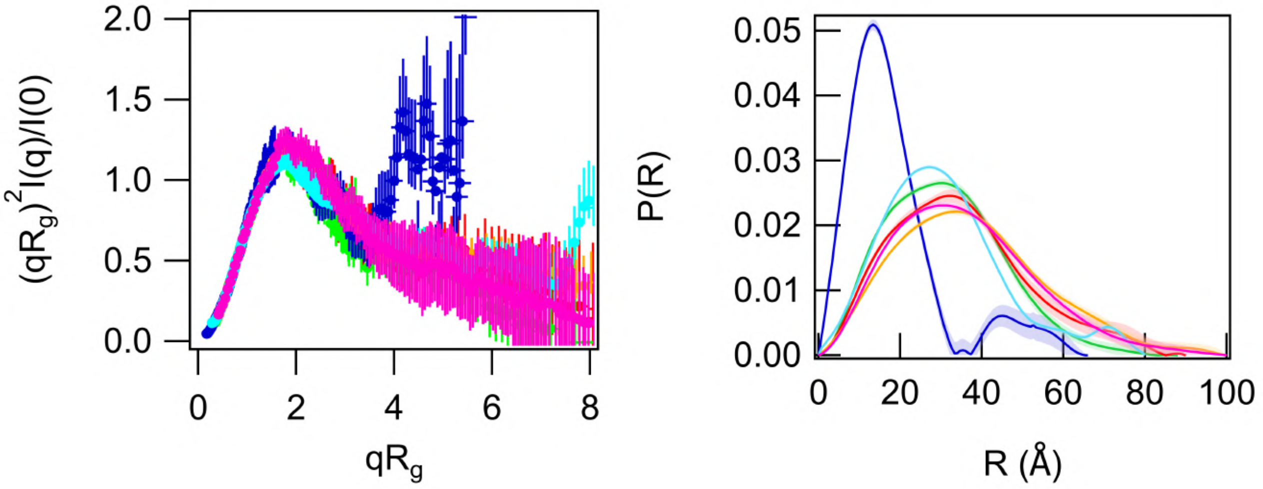

Figure S8: **Dimensionless Kratky Plot and *P(r)* MexR-PII.** The graphs show the Dimensionless Kratky plot of the data set of MexR-PII and distances of distribution of the data set acquired. The *P(r)* is calculated with the ATSAS software and normalised by the area, and the light area represents the uncertainty that ATSAS software give for the *P(r)*. Colorcode: SAXS of fully protonated complex (pink), SANS of fully protonated in 0% (yellow) and 79 % (cyan) D_2O buffers, and SANS of the dMexR-PII complex in 0 % (red), 56 % (green) and 89 % (navy) D_2O buffers. Note that dMexR-PII in 89% D_2O is not recorded at match point and therefore contains both the contribution from the DNA and the protein.

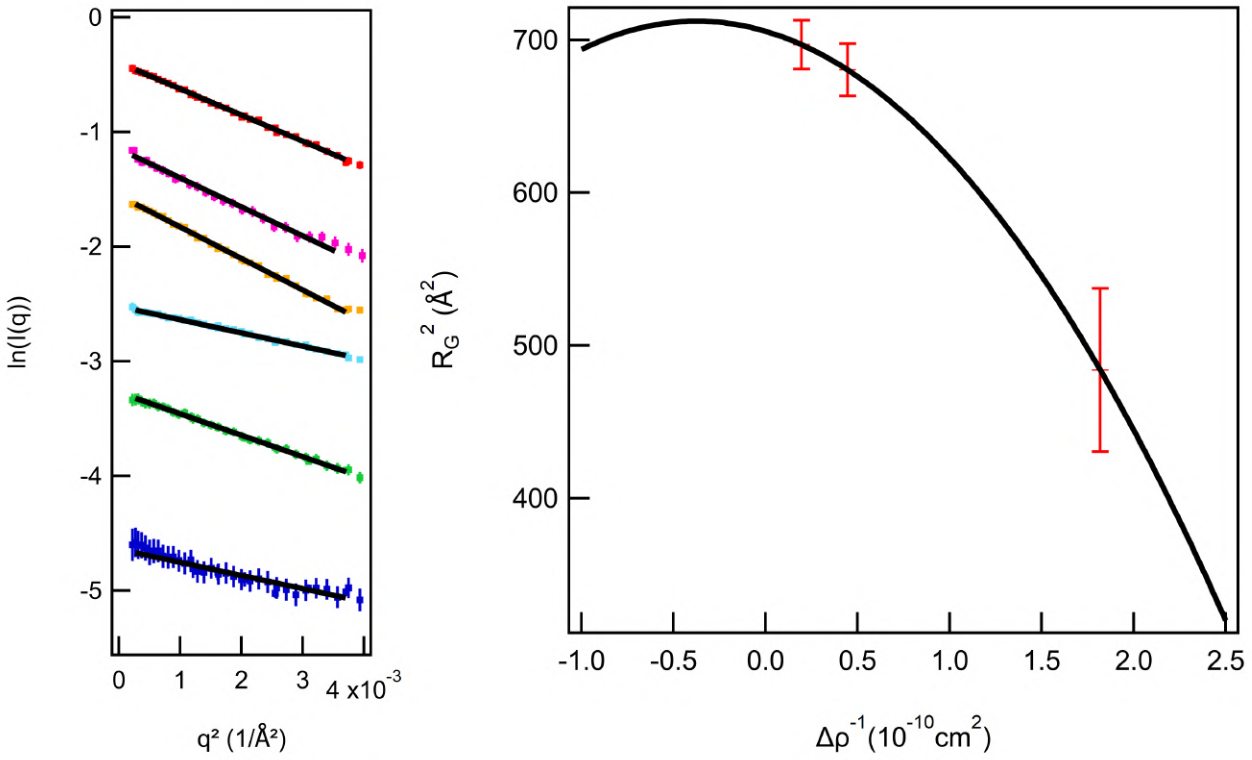

Figure S9: **Guinier fit for all the data set for MexR-DNA performed with the NCNR macro for IGOR and Stuhrmann plot.** Left plot: Color code: SAXS of fully protonated complex (pink), SANS of fully protonated in 0% (yellow) and 79 % (cyan) D_2O buffers, and SANS of the dMexR-PII complex in 0 % (red), 56 % (green) and 89 % (navy) D_2O buffers. The ranges and the results of the linear fits are shown in table S2. Right plot: Stuhrmann plot of the set of data with partially deuterated protein with its parabolic fit $R_g^2 = R_v^2 + \frac{\alpha}{\Delta\rho} - \frac{\beta}{\Delta\rho^2}$ used as a guide for the eyes. Since $\beta \neq 0$ this tell us that the components do not have the same center of mass, and since $\alpha < 0$ this indicates that the more dense component (*ie* the protein) is closer to the center of mass (40). Both of these conclusions agree with *ab-initio* and molecular models as presented in Figure 4B.

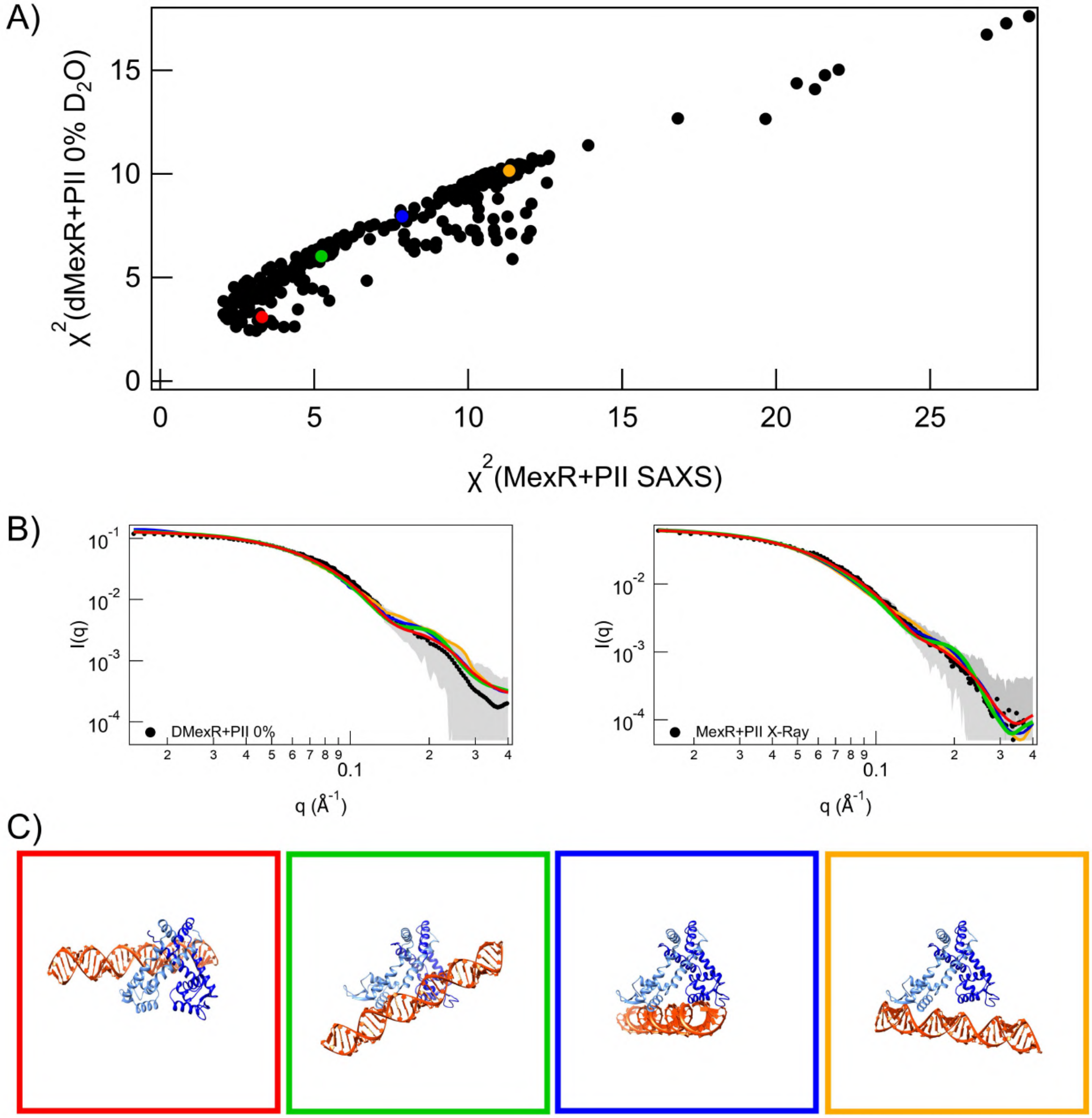

Figure S10: **H-Dock Docking results.** A) Scatter plot of the χ^2 of dmexR PII 0% D_2O over the χ^2 of MexR PII SAXS data. It is possible to notice a linear trend, but still the χ^2 are all above 2. B) The graphs represent the overlap between the experimental data (black dots) and the simulated curves. The structures were generated with the docking, and they are listed below in C). The colours of the curves correspond to the colours of the frames of the structures. C) Four examples of structures generated with H-Dock. For clarity, the orientation of the MexR is the same in all the pictures

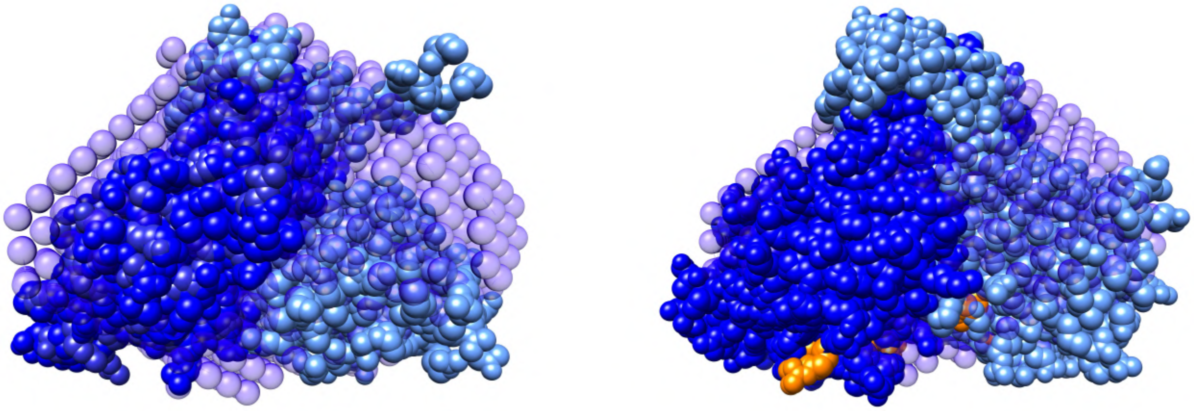

Figure S11: DAMFILT *ab-initio* model (beads) superposed with the 4 structures of 1LNW (left) and the representatives of the best-fitting ensemble of 21 structures representing apo-MexR shown in figure 4D) (right).

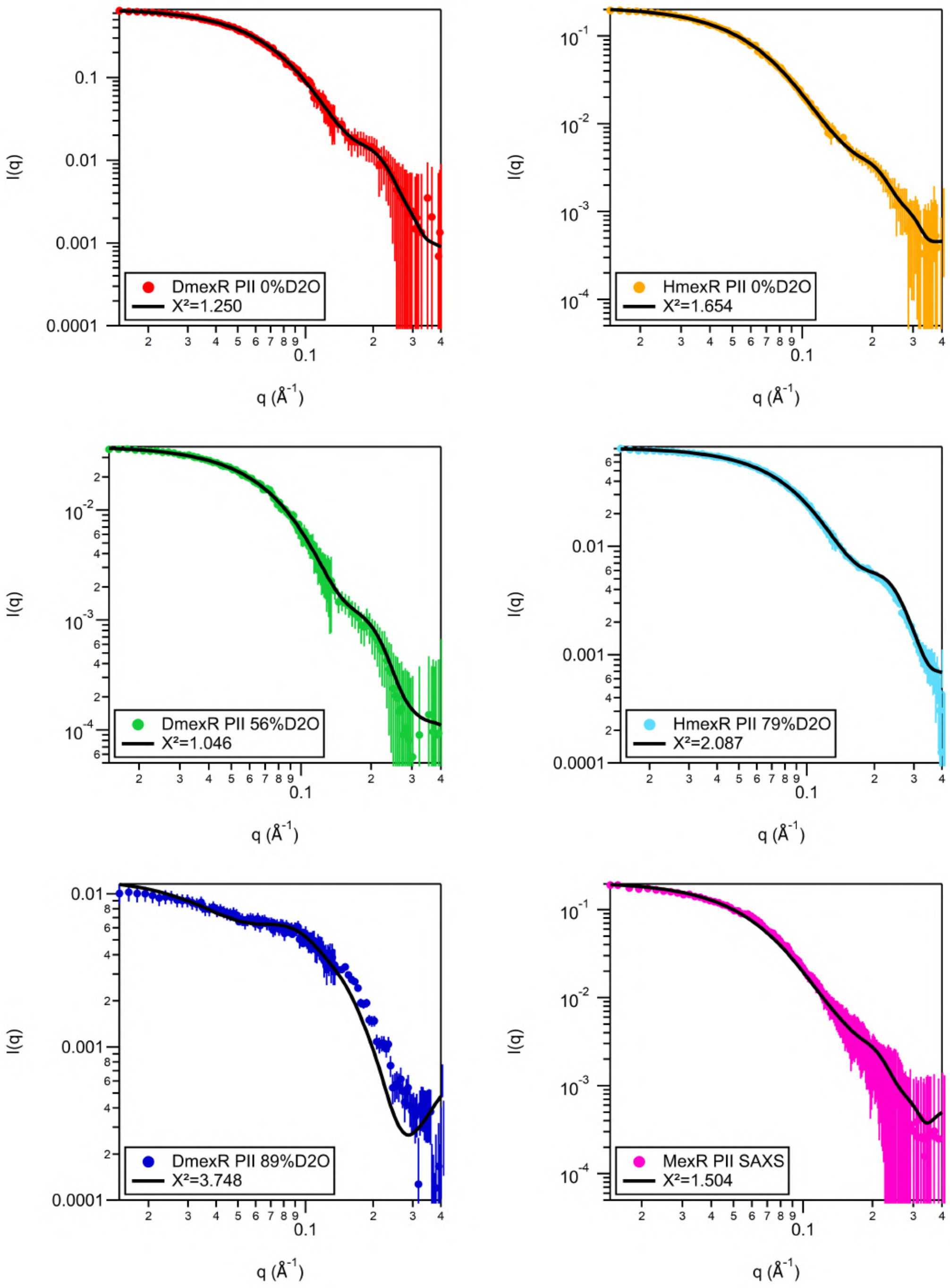

Figure S12: **SANS data set of MexR-PII complex supplemented with laboratory SAXS data of fully-protonated MexR-PII with CRYSOL/N fit.** The graphs show the reduced data of the experiment at D22 of the MexR bound with PII DNA. The black lines passing through the experimental point is the CRYSOL/N fit of the structure shown in figure 3B. Colorcode: SAXS of fully protonated complex (pink), SANS of fully protonated in 0% (yellow) and 79 % (cyan) D_2O buffers, and SANS of the dMexR-PII complex in 0 % (red), 56 % (green) and 89 % (navy) D_2O buffers. The experimental data are the same as in Figure S6 even they have discrepancy in intensity and q-range.
